# Antioxidant, anti‐inflammatory, and anticancer function of *Engleromyces goetzei* Henn aqueous extract on human intestinal Caco‐2 cells treated with t‐BHP

**DOI:** 10.1002/fsn3.3335

**Published:** 2023-04-06

**Authors:** Ni Jun, Cheng Yi‐Ting, Gao Yu‐Ting, Zhao Cheng‐Fa, Li Li‐Juan, She Rong, Yang Xiao‐yan, Xiao Wen, Yang Xu

**Affiliations:** ^1^ Institute of Natural Antioxidants and Antioxidant Inflammation Dali University Dali 671003 China; ^2^ Institute of Eastern‐Himalaya Biodiversity Research Dali University Dali Yunnan China; ^3^ Center for Cultural Ecology in Northwest Yunnan Dali Yunnan 671003 China; ^4^ Yunling Back‐and‐White Snub‐Nosed Monkey Observation and Research Station of Yunnan Province Dali Yunnan 671003 China; ^5^ Laboratory of Environmental Biomedicine Central China Normal University 430079 Wuhan China

**Keywords:** anticancer, anti‐inflammatory, antioxidative stress, biocompatibility, *Engleromyces goetzei* Henn (*Eg*H), LC–MS analysis, obesity‐induced diseases

## Abstract

High body mass index (high BMI, obesity) is a serious public health problem, and “obesity‐induced oxidative stress, inflammation, and cancer” have become modern epidemic diseases. We carried out this study to explore a functional beverage that may protect against obesity‐induced diseases. The *Engleromyces goetzei* Henn herbal tea is such a candidate. For this study, we carried out LC–MS analysis of *E. goetzei* Henn aqueous extract (*Eg*H‐AE); then used the Caco‐2 cell line for the model cells and treated the cells with t‐BHP to form an oxidative stress system. An MTT assay was used for testing the biocompatibility and cytoprotective effects; reactive oxygen species and malondialdehyde determination was used for evaluating the antioxidative stress effect; TNF‐α and IL‐1β were used for observing the anti‐inflammatory effect, and 8‐OHdG for monitoring anticancer activity. The results of this study demonstrate that the *Eg*H‐AE has very good biocompatibility with the Caco‐2 cell line and has good cytoprotective, antioxidant, anti‐inflammatory, and anticancer properties. It is clear that *Eg*H‐AE, a kind of ancient herbal tea, may be used to develop a functional beverage that can be given to people with a high BMI to protect against obesity‐induced diseases.

## Graphical abstract

*Engleromyces goetzei* Henn (*Eg*H) is a natural fungus that has been used as a traditional edible and medicine for long time in Southwest China. Our study found *Eg*H aqueous extract (*Eg*H‐AE) has very strong activities on antioxidant and anti‐inflammation. At the same time, we also found *Eg*H‐AE has good biocompatibility and cell protective function, so it is biosafe, *Eg*H‐AE may have the prospect of developing into functional beverage.

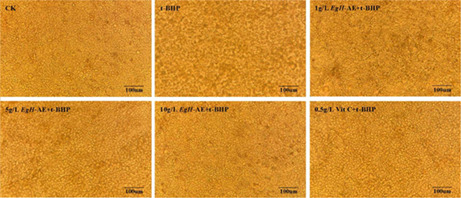

## 1 INTRODUCTION

With the change in living environment, people are now more and more easily able to obtain food, especially high‐calorie food. At the same time, due to the increasing pace of lifestyle, fast food has become the food of choice for most people, and these foods are usually rich in calories, sugar, and fat, coupled with factors such as the constant reduction of exercise time, obesity is becoming more common in the population (Brewer & Balen,  2010).

Currently, obesity is one of the most common public health problems facing the world today and has been listed as one of the top 10 health risk factors by the World Health Organization. According to global deaths for all ages, a high body mass index (obesity) is fifth among all risk factors and was responsible for the death of about 5,019,360 people in 2019 (The Institute for Health Metrics and Evaluation, 2020). The prevalence of obesity is also very high. Taking China as an example, 16.4% of adults are obese, a population of 150 million, while the overweight adult population (including obese patients) is close to 500 million, or 50.7% of the adult population (Xiao & Yang, 2022). At present, “obesity‐induced oxidative stress” (Lin et al., 2019; Shen et al., 2020; Taherkhani et al., 2021), “obesity‐induced inflammation” (Battineni et al., 2021; Kim & Lee, 2021; Kwaifa et al., 2020; Land Lail et al., 2021; Sudhakaran & Doseff, 2020), and “obesity‐induced cancer” (Annett et al., 2020; Kern et al., 2018; Ostrand‐Rosenberg, 2021; Rajesh & Sarkar, 2021; Yau et al., 2018) have become very worrying modern diseases and pathological changes and pose a major challenge for global public health.

In terms of pathogenesis, oxidative stress is usually an upstream event of inflammation and cancer (Reuter et al., 2010). Reactive oxygen species (ROS) can induce the synthesis and release of inflammatory factors such as TNF‐α and IL‐1β, by activating the NFκB signaling pathway. In addition, high levels of ROS in cells attack guanine bases in DNA easily and form 8‐hydroxydeoxyguanosine (8‐OHdG), and 8‐OHdG can induce the occurrence of various cancers. Therefore, in the face of the above diseases, in addition to clinical treatment, appropriate use of antioxidant functional foods could also form a very important strategy for the prevention and treatment of obesity‐induced diseases.

At this stage, antioxidant functional foods are mainly derived from plants, such as grapes, blueberries, kiwifruit, green tea, etc.; these foods are rich in antioxidants, such as blueberries rich in anthocyanins, kiwi fruit rich in vitamin C, green tea rich in tea polyphenols, and these substances play a significant role in maintaining the health of the body and slowing down the oxidation of the body (Ramarathnam et al., 1995). At the same time, edible and medicinal fungi are a kind of functional food that is rich in nutrients and has vigorous antioxidant activity; at present, edible and medicinal dual‐use fungi have become another important source of antioxidant functional food (Mwangi et al., 2022).

*Engleromyces goetzei* Henn. (*Eg*H) (Fungal, Ascomycetes, Hypocreaceae, Hypocrea), the shape is irregularly spherical, 2–20 cm in diameter, nearly smooth in appearance with fine black spots, the whole is pink or light flesh, anaphase becomes milky white, grayish to brown, the inside is light red to gray–white, solid, hard after drying, interwoven by short and irregular branched mycelium, containing a large number of spherical oil droplets, mature bamboo fungus surface is often uneven, or with irregular protrusions, the fungus meat has a slightly bitter taste after chewing (Yunnan Institute of Botany, 1975). In China, *Eg*H parasitic on high mountain bamboo poles and matures in the rainy season from July to August, this fungus is mainly distributed between 25–30° N and 98–103° E, that is, Northwest Yunnan, Southwest Sichuan, and Southeast Tibet. The terrain of this region is steep, and many mountains are arranged in parallel in a north–south direction. It is one of the regions with the highest biodiversity in Asia and even in the world. *Eg*H is also a native functional food and medicine with a long tradition of use in Yunnan, Sichuan, Tibet, and other places. It is often used by local residents to make tea with boiling water and is used to treat infection, inflammation, and cancer (Jikai et al., 2002; Wang, Zhang, Li, et al., 2015). An earlier experimental study found that *Eg*H has a broad‐spectrum antibacterial effect (Yunnan Institute of Botany, 1975). Zhang et al. (2019) found experimentally that *Eg*H has an antiproliferation effect. Wang's study (Wang, Zhang, Wang, et al., 2015) found that *EgH* inhibits cholesterol ester transfer protein. These are the only studies found in the literature on the biological activity of *Eg*H based on cellular and in vivo experiments.

We, therefore, considered exploring whether the traditional remedy using *Eg*H tea can be used to prevent and treat obesity‐induced oxidative stress, inflammation, and cancer. In this study, we investigated the antioxidant, anti‐inflammatory, and anticancer effects of *E. goetzei* Henn aqueous extract (*Eg*H‐*AE*) on Caco‐2 cells treated with t‐BHP. The results support the possibility that *Eg*H‐*AE* may be developed as a functional beverage to protect the health of obese patients.

## 2 MATERIALS AND METHODS

### 2.1 Chemicals and reagents

Bi‐distilled deionized water system (Laboratory Water Purification System; Shanghai Hitech Instrument Co., Ltd), rotary evaporator (RE‐2000A Rotary Evaporator; Shanghai Yarong Biochemical Instrument Factory), ultrasonic extractor (SB25‐12DTD Ultrasonic Cleaner; Ningbo Xinzhi Biotechnology Co., Ltd.), and freeze dryer (SCIENTZ‐10N Freeze Dryer; Ningbo Xinzhi Biotechnology Co., Ltd.) were used in this study, High‐speed centrifuge (Hunan Xiangyi Experiment Equipment Co., Ltd.), Vortex mixer (Haimen Kylin‐bell Lab Instruments Co., Ltd.), Microporous membrane filters (0.22 μm; Tianjin Jinteng Experiment Equipment Co., Ltd.), Thermo Vanquish (Thermo Fisher Scientific), Thermo Q Exactive Focus (Thermo Fisher Scientific).

The chemicals used were all of high purity or analytical reagent grade. 2‐Chloro‐l‐phenylalanine, Folin–Ciocalteu reagent, phenol, petroleum ether, ethyl acetate, *n*‐butanol, 95% ethanol, Na_2_CO_3_, NaNO_2_, Al(NO_3_)_3_, NaOH, and H_2_SO_4_ were obtained from Sinopharm Chemical Reagent Co. Ltd. 2,2‐diphenyl‐1‐pycrilhydracyl acid (DPPH) was purchased from Tixiai Chemical Industry Development Co., Ltd. LC–MS grade acetonitrile (ACN) was purchased from Fisher Scientific. Formic acid was obtained from TCI. Ammonium formate was obtained from Sigma‐Aldrich. Ultrapure water was generated using a Milli‐Q system. 2,2′‐Azino‐bis (3‐ethylbenzthiazoline‐6‐sulfonic acid) (ABTS) and tert‐butyl hydroperoxide (t‐BHP) were obtained from Shanghai McLean Biochemical Technology Co., Ltd. Ethyl acetate and glacial acetic acid were purchased from Guangdong Chemical Reagent Engineering Technology R&D Center, China. DMEM high glucose medium came from Gibco, USA. Ascorbic acid (Vit. C), anhydrous sodium acetate, and anhydrous ethanol were purchased from Beijing Solarbio Science & Technology Co., Ltd. 3‐(4,5‐Dime thylthiazol‐2‐yl)‐2.5‐diphenyltetrazolium bromide (MTT) kit and 8‐hydroxy‐2 deoxyguanosine (8‐OHdG) kit were purchased from Shanghai Enzyme Link Biotechnology Co., Ltd. Malondialdehyde (MDA) kit was from Nanjing Jiancheng Bioengineering Institute. Interleukin‐1β (IL‐1β) and tumor necrosis factor‐α (TNF‐α) kits were purchased from Wuhan Boster Bioengineering Co., Ltd. ROS kit was from Jiangsu Jingmei Biological Technology Co., Ltd.

### 2.2 Preparation of *Engleromyces goetzei* Henn extracts

**FIGURE 1:**
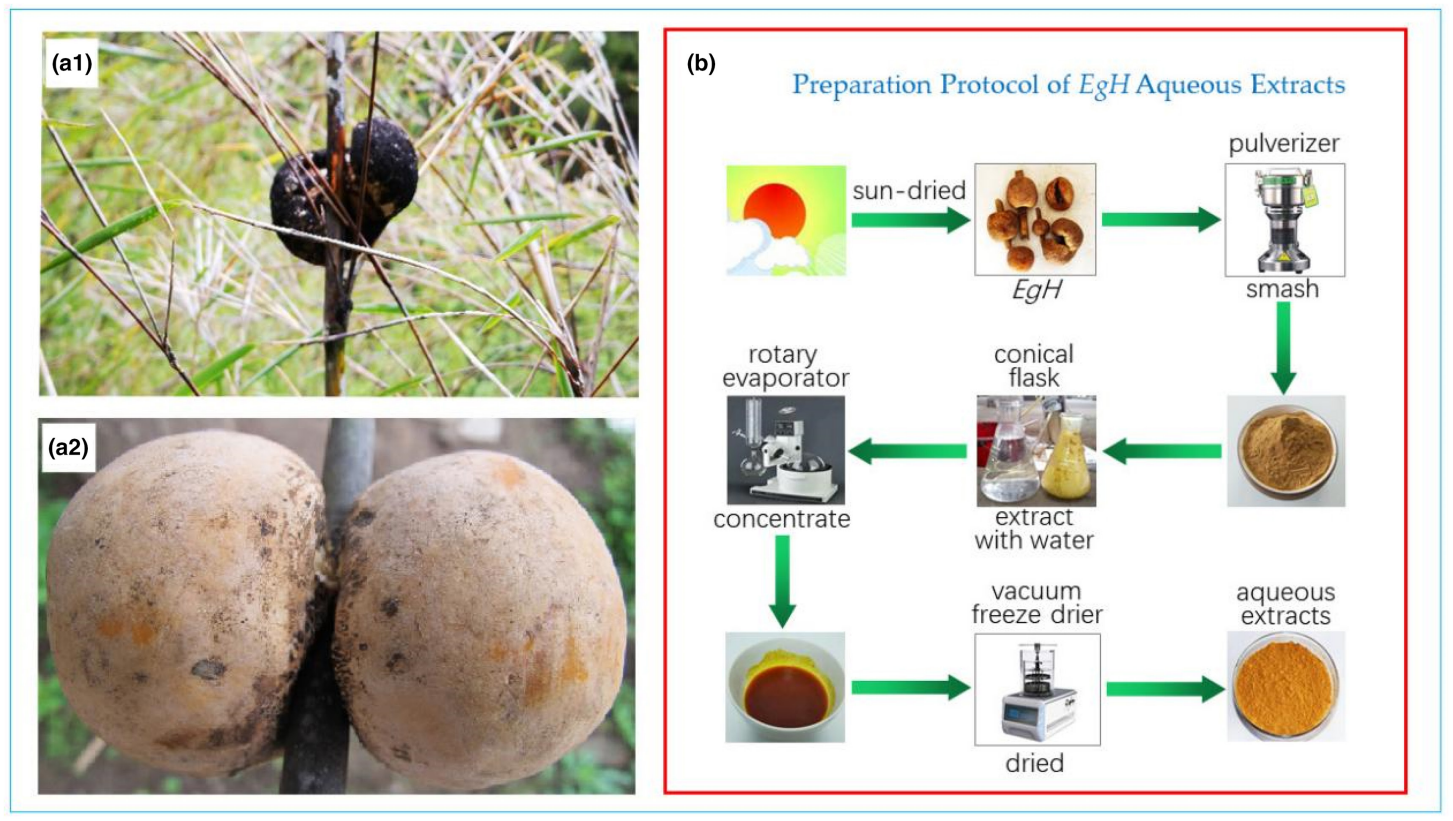
Photographs of *Eg*H and the sample extraction procedure. a1 and a2 (our image), (b) Preparation protocol for *Eg*H extraction, the *Eg*H images are ours.

Natural *E. goetzei* Henn (*Eg*H) were harvested from Yunling‐Lasha Mountain Nature Reserve, Yunnan Province, China during the summer of 2021. Figure 1 shows a sample image of *Eg*H and the preparation protocol of the *Eg*H extracts.

The sun‐dried *Eg*H sample was smashed and passed through a 100‐mesh sieve. Place 50 g of *Eg*H powder into a 500 mL conical flask, and add 500 mL of petroleum ether according to a material‐to‐liquid ratio of 1:10. At 20°C, 60% power, 40 Hz ultrasonic extraction for 1 h. After standing in the dark for 24 h, the supernatant was decanted using a pipette, suction filtered, and the filtrate concentrated by rotary evaporation. The remains were freeze‐dried to obtain the petroleum ether extract of *Eg*H. The filter residue was dried at 40°C, and the above operation was repeated using ethyl acetate, *n*‐butanol, 95% ethanol, and distilled water to successively extract filter residue, the ethyl acetate extract, *n*‐butanol extract, 95% ethanol extract, and aqueous extract of *Eg*H were obtained, respectively, and stored at 4°C for future use.

### 2.3 DPPH assay

The DPPH radical scavenging assay was performed according to a previous report (Brand‐Williams et al., 1995). The *Eg*H aqueous extract (*Eg*H‐AE) was diluted with absolute ethanol to form different concentrations of solvent (15, 10, 5, 2.5, 1.25, and 0.625 mg/mL). 50 mg/L DPPH absolute ethanol solvent was blended in the ratio of 1:1 and left to stand at 37°C for 30 min in the dark. Absolute ethanol was used as the control. Absorbance of the reaction mixtures was recorded at 517 nm. Five replicates, one sample control, and one blank control experiment were set up for each concentration. In addition, a standard curve was drawn with a DPPH of 5–100 mg/L. The DPPH radical scavenging activity of the *Eg*H extract was calculated following the equation:

$$\text{DPPH radical scavenging activity}=\frac{\mathrm{Abs}\;\text{of control}-\mathrm{Abs}\;\text{of sample}\;}{\mathrm{Abs}\;\text{of control}}\times 100\%.$$

### 2.4 ABTS assay

The ABTS radical scavenging assay was performed according to a previous report (Miller et al., 1993). The *Eg*H‐AE was diluted with absolute ethanol to form different concentrations of solvent (15, 10, 5, 2.5, 1.25, and 0.625 mg/mL). ABTS radical ethanol solvent was blended with each sample in the ratio of 1:1 and left to stand at room temperature for 6 min. Absolute ethanol was used as the control. Absorbance of the reaction mixtures was recorded at 734 nm. Six replicates, one sample control, and one blank control experiment were set up for each concentration. The ABTS radical scavenging activity of the *Eg*H extract was calculated as follows:

$$\text{ABTS radical scavenging activity}=\frac{\mathrm{Abs}\;\text{of control}-\mathrm{Abs}\;\text{of sample}\;}{\mathrm{Abs}\;\text{of control}}\times 100\%.$$

### 2.5 Measurement of total phenolic, flavonoid, and polysaccharide content

The total phenolic content was quantified using Folin–Ciocalteu reagent and gallic acid standard (Ainsworth & Gillespie, 2007). Briefly, 1 g/mL of *Eg*H‐AE was mixed in a test tube containing 5 mL 10% Folin–Ciocalteu reagent. The mixture was then allowed to react for 3–8 min, after which 4 mL of 7.5% Na_2_CO_3_ was added. The mixture was then placed in a dark room for 1 h, and the absorbance was measured at 765 nm. The total phenolic content is expressed as gallic acid equivalents (μg GAE/mg).

To analyze total flavonoid content in *Eg*H‐AE (Zhishen et al., 1999), 1 g/mL of each sample was mixed with 300 μL of 5% (w/v) NaNO_2_ and 300 μL of 10% Al(NO_3_)_3_, The mixture was then allowed to react for 6 min, after which 1 mL of 1 mol/L NaOH and 3.4 mL of 30% ethyl alcohol was added. The mixture was then placed in a dark room for 15 min, and the absorbance was measured at 510 nm. The total flavonoid content is expressed as rutin equivalents (μg RE/mg).

To analyze the total polysaccharide content of *Eg*H‐AE (Dubois et al., 1951), 1 g/mL of each sample was mixed with 1 mL of 5% phenol and 5 mL of 1.84 g/mL H_2_SO_4_. The mixture was then placed in a dark room for 30 min, and the absorbance was measured at 490 nm. The total polysaccharide content is expressed as glucose equivalents (μg GE/mg).

### 2.6 LC–MS analysis

LC–MS was performed in electrospray ionization (ESI) mode with a liquid chromatography instrument (U3000; Thermo Fisher) combined with a mass spectrometer (QE Plus; Thermo Fisher). Accurately add 300 μL of water prepared with 2‐chloro‐l‐phenylalanine (4 ppm) solution for redissolution *Eg*H‐*AE*. The *Eg*H‐*AE* was centrifuged at 17,435 *g* at 4°C for 10 min and filtered through a 0.22 μm membrane. An ACQUITY UPLC* HSS T3 chromatographic column (1.8 μm, 2.1 × 150 mm; Waters) was used. The column was maintained at 40°C. The flow rate and injection volume were set at 0.25 mL/min and 2 μL, respectively. For LC‐ESI (+)‐MS analysis, the mobile phases consisted of (B2) 0.1% formic acid in acetonitrile (v/v) and (A2) 0.1% formic acid in water (v/v). Separation was conducted under the following gradient: 0–1 min, 2% B2; 1–9 min, 2%–50% B2; 9–12 min, 50%–98% B2; 12–13.5 min, 98% B2; 13.5–14 min, 98%–2% B2; 14–20 min, 2% B2. For LC‐ESI (−)‐MS analysis, the analytes were carried out with (B3) acetonitrile and (A3) ammonium formate (5 mM). Separation was conducted under the following gradient: 0–1 min, 2% B3; 1–9 min, 2%–50% B3; 9–12 min, 50%–98% B3; 12–13.5 min, 98% B3; 13.5–14 min, 98%–2% B3; 14–17 min, 2% B3. Mass spectrometric detection of metabolites was performed on Q Exactive Focus (Thermo Fisher Scientific) with ESI ion source. Simultaneous MS1 and MS/MS (Full MS‐ddMS2 mode, data‐dependent MS/MS) acquisition were used. The parameters were as follows: sheath gas pressure, 30 arb; aux gas flow, 10 arb; spray voltage, 3.50 and −2.50 kV for ESI(+) and ESI(−), respectively; capillary temperature, 325°C; MS1 range, m/z 100–1000; MS1 resolving power, 70,000 FWHM; the number of data‐dependent scans per cycle, 3; MS/MS resolving power, 17,500 FWHM; normalized collision energy, 30 eV; dynamic exclusion time, automatic. The identification of the chemical composition was first confirmed by the exact molecular weight (molecular weight error ≤30 ppm), and then the Human Metabolome Database (HMDB) (http://www.hmdb.ca, accessed 5 August 2022), METLIN (http://metlin.scripps.edu, accessed 10 August 2022), MassBank (http://www.massbank.jp/, accessed 5 August 2022), LipidMaps (http://www.lipidmaps.org, accessed 5 August 2022), mz Clound (https://www.mzcloud.org, accessed 5 August 2022), and BioNovoGene self‐built standard product database confirmed that the chemical composition was obtained.

### 2.7 Cell culture

Caco‐2 cells (human colon adenocarcinoma cells) were purchased from Kunming Cell Bank of Type Culture Collection, Chinese Academy of Sciences. The cells were inoculated into a 75 cm^2^ cell culture flask containing 15 mL culture medium, placed in a 37°C, 5% CO_2_, and high humidity incubator, and the culture medium was changed every 24 h. The culture medium comprised 84% DMEM high glucose medium, 15% fetal bovine serum, 1% penicillin (1000 U/mL), and 1% streptomycin (1000 μ g/mL). The cells were subcultured after 80%–90% of the bottom of the bottle.

### 2.8 MTT assay

Also called a cell viability assay, this was carried out by the 3‐(4,5‐dimethylthiazol‐2‐yl)‐2,5‐diphenyltetrazolium bromide (MTT) method (Mosmann, 1983). Briefly, the Caco‐2 cells were seeded into a 96‐well cell culture plate with 1 × 10^5^ cells/well, and treated with different concentrations of *Eg*H‐AE (0.05, 0.1, 0.2, 0.4, 0.8, 1.6, 3.2, 6.4, and 12.8 mg/mL) for 24 h at 37°C under 5% CO_2_. The cell viability (%) was determined using a commercial kit (Cat. No. 16H12B56; Shanghai Enzyme Link Biotechnology Co., Ltd.). All samples were prepared according to the instructions for the use of the MTT kit. Absorbance was measured at 490 nm using a microplate reader (DNM‐9602G; Beijing Pulang New Technology Co., Ltd.).

The concentration of Caco‐2 cells was adjusted to 5 × 10^5^ cells/mL, inoculated 2.0 mL cell suspension in each well of the 6‐well plate, and placed in the incubator for culture, changing the solvent every 12 h. After 2 days, discard the cell culture medium, add 1 mL PBS to each well to clean it, and repeat twice. The Caco‐2 cells were then preprotected for 12 h with different concentrations (0.0, 1.0, 5.0, and 10 mg/mL) *Eg*H‐AE and 0.5 mg/mL Vit C. After that, the cells were treated with 2 μmoL/mL t‐BHP. The *Eg*H‐AE, t‐BHP, and Vit C were diluted with DMEM medium. Each group was equipped with six experimental holes. After 5 h, the cell viability (%) was determined using a commercial kit (Cat. No. 16H12B56; Shanghai Enzyme Link Biotechnology Co., Ltd.). All samples were prepared according to the instructions for the use of the MTT kit.

### 2.9 Detection of ROS, MDA, 8‐OHdG, TNF‐α, and IL‐1β

The concentration of Caco‐2 cells was adjusted to 5 × 10^5^ cells/mL, inoculate 2.0 mL cell suspension in each well of 6 well plate, place it in the incubator for culture changing the solvent every 12 h. After 2 days, discard the cell culture medium, add 1 mL PBS to each well to clean it, and repeat twice. The Caco‐2 cells were then preprotected for 12 h with different concentrations (0.0, 1.0, 5.0, and 10 mg/mL) *Eg*H‐AE and 0.5 mg/mL Vit C. After that, the cells were treated with 2 mmol t‐BHP. The *Eg*H‐AE, t‐BHP, and Vit C were diluted with DMEM medium. Each group was equipped with six experimental holes. After 5 h, sample twice from each hole taking 100 μL each time into 1.5 mL centrifuge tubes. These were then sealed and stored at −20°C for later determination of ROS, MDA, TNF‐α, and IL‐1β, 8‐OHdG. All samples were prepared according to the instructions for the kits, and absorbance was measured using a microplate reader (DNM‐9602G; Beijing Pulang New Technology Co., Ltd).

Reactive oxygen species was measured at 450 nm using the ROS kit (Cat. No. 202204; Shanghai Enzyme Link Biotechnology Co., Ltd). MDA was measured at 532 nm using the MDA kit (Cat. No. 20220221; Nanjing Jiancheng Bioengineering Institute). 8‐OHdG, TNF‐α, and IL‐1β were measured at 450 nm using the 8‐OHdG kit (Cat. No 02/2022; Shanghai Enzyme Link Biotechnology Co., Ltd.), and TNF‐α and IL‐1β kits (Cat. No. 2391811208, and Cat. No. 1141816208; Wuhan Boster Bioengineering Co., Ltd.).

### 2.10 Statistical analyses

All data in this study were analyzed by one‐way ANOVA, followed by an LSD test, the LSD test at 5% precision level was used to ascertain the significance and nonsignificance of different treatment and control groups. Data were processed with GraphPad Prism 7 software and results were expressed as the mean and standard error of the mean (mean ± SD).

## 3 RESULTS AND DISCUSSION

### 3.1 Free radical scavenging activity of *Eg*H extracts

**FIGURE 2:**
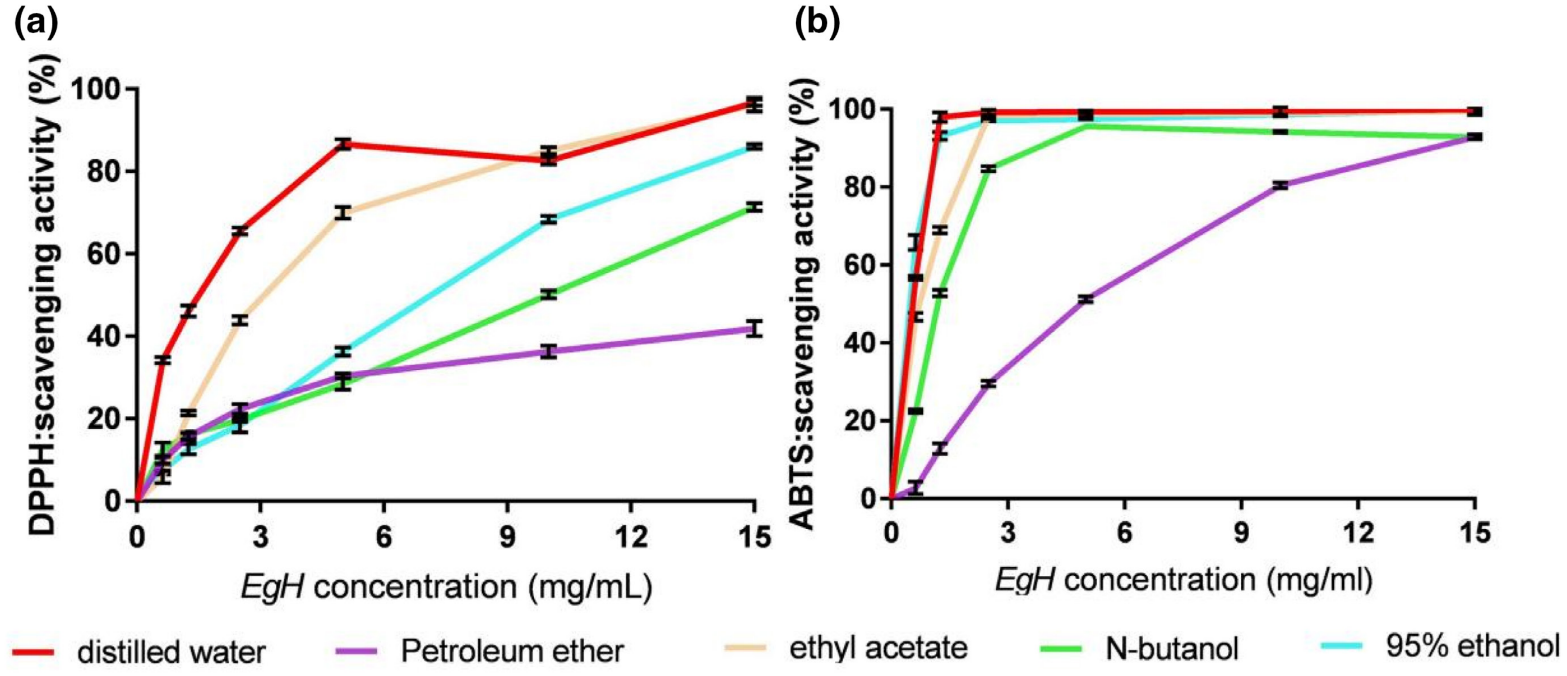
Antioxidant activity of *Eg*H extracts. (a) DPPH scavenging activity of *EgH* with different solvents; (b) ABTS scavenging activity of *Eg*H with different solvents.

In this study, we used DPPH and ABTS radical scavenging assays to evaluate the antioxidant activity of the *Eg*H extracts with different solvents. The results are presented in Figure 2 and Table 1. As shown in Figure 2, the *Eg*H extracts made with five solvents (petroleum ether, ethyl acetate, *n*‐butanol, 95% ethanol, and distilled water), scavenge DPPH and ABTS in a concentration‐dependent manner. Of the five extracts, the *Eg*H‐AE made using distilled water demonstrates the best antioxidant activity. From Table 1, it is also clear that all of the five extracts made with the five different solvents have relatively strong antioxidant capacity, compared with the other extracts listed in Table 1, with the best extract being *Eg*H‐AE.

**TABLE 1:** Comparisons of the IC_50_ obtained by DPPH and ABTS assay with references.

Study in	Samples for extracting	IC_50_ (μg/mL)		Data source
		DPPH assay	ABTS assay	
China, 2022	*Eg*H*—*aqueous extract	1.30	0.55	This study
	*Eg*H—95% ethanol extract	5.85	0.38	
	*Eg*H—ethyl acetate extract	2.95	0.71	
	*Eg*H—*n*‐butanol extract	8.84	1.15	
	*Eg*H—petroleum ether extract	25.49	4.29	
Japan, 2019	Vin tea polyphenols from Hunan	4.51	—	Xie et al. (2019)
	Vin tea polyphenols from Guizhou	4.06	—	
	Vin tea polyphenols from Guangxi	4.31	—	
	Dihydromyricetin	3.24	—	
Italy, 2020	Tomato pomace from industry 1	57.9	—	Abbasi‐Parizad et al. (2020)
	Tomato pomace from industry 2	92.7	—	
Italy, 2020	*Fucus vesiculosus*—assay B200214	614	—	Corsetto et al. (2020)
	*Fucus vesiculosus*—assay B290814	608	—	
Portugal, 2020	Chestnut shells—sample no. 1	56.5	66.4	Lameirão et al. (2020)
	Chestnut shells—sample no. 2	59.9	69.6	
	Chestnut shells—sample no. 3	56.7	64.4	
	Chestnut shells—sample no. 4	44.1	65.3	
	Chestnut shells—sample no. 5	63.7	50.5	
China, 2021	Kiwi leaves—ultrasound extract	13.67	10.88	Lv et al. (2021)
	Kiwi leaves—maceration extract	17.59	13.67	
Costa Rica, 2019	Keitt skin	11.93	—	Navarro et al. (2019)
	Keitt flesh	17.78	—	
	T. Atkins skin	9.97	—	
	T. Atkins flesh	22.51	—	
Italy, 2022	Lyophilized olive mill wastewater	95	19	Spizzirri et al. (2022)
China, 2018	Peptide‐A: GAERP	3730	100	Tao et al. (2018)
	Peptide‐B: GEREANVM	1870	50	
	Peptide‐C: AEVG	2300	70	
China, 2019	Peptide‐A: VPR	4610	4010	Pan et al. (2019)
	Peptide‐B: IEPH	770	1300	
	Peptide‐C: LEEEE	80	160	
	Peptide‐D: EEEQ	150	180	
China, 2020	Collagen peptide‐1: DGPEGR	4240	—	Wang et al. (2020)
	Collagen peptide‐2: GPEGPMGLE	590	—	
	Collagen peptide‐3: EGPFGPEG	370	—	
	Collagen peptide‐4: YGPDGPTG	1760	—	
Italy, 2019	Grape leaves—water crude extract	150	—	Ferhi et al. (2019)
	Grape leaves—ethanolic crude extract	90	—	
China, 2020	Butylated hydroxytoluene (BHT)	30	10	Deng et al. (2020)
China, 2020	Rape bee pollen—crude extract	76.87	—	Zhang et al. (2020)
	Rape bee pollen—flavonoids	34.19	—	
	Rape bee pollen—phentolamines	6.41	—	
China, 2020	*Plantago asiatica*—dichloromethane extract	351	——	Dong et al. (2020)
	*Plantago asiatica*—ethyl acetate extract	160	—	
	*Plantago asiatica*—methanol extract	153	—	
Portugal, 2018	Saco sweet cherry—total extract	21.88	—	Gonçalves et al. (2018)
	Saco sweet cherry—colored fraction	31.39	—	
	Saco sweet cherry—noncolored fraction	210.86	—	

In the latter experiments of this study, we used only *Eg*H‐AE as our test sample, because: (1) in the earlier experiments we found that the antioxidant effect of *Eg*H‐AE had the best effect of the five extracts; (2) this study aimed to simulate the general preparation process of the beverage to be drunk, since this fungus has been used for a long time as a herbal tea in Western Yunnan, China; (3) to obtain the most accurate results of antioxidant, anti‐inflammatory, and antigenotoxic activity for the extract, it is best that the samples are not contaminated by solvents, and are nontoxic.

### 3.2 Chemical composition of *Eg*H‐AE

The total phenolic, flavonoid, and polysaccharide content in *Eg*H‐AE is shown in Table 2. It is clear that the main chemical components in *Eg*H‐AE are polysaccharides. This means that the polysaccharides in *Eg*H‐AE possibility perform the main antioxidant activity. This finding is consistent with other studies (Ren et al., 2015; Wang et al., 2018). In other words, the main antioxidant components of *Eg*H‐AE are polysaccharides. In fact, fungal polysaccharides are widely distributed in the cell walls of fungi. Medicinal fungal polysaccharides are widely known as “biological response modifiers”.

**TABLE 2:** Total phenolic, flavonoid, and polysaccharide content of *Eg*H‐AE.

Chemical composition	Measurement (mean ± SD)
Total phenolic content (TPC) (μg GAE/mg)	6.155 ± 0.239
Total flavonoid content (TFC) (μg RE/mg)	5.758 ± 0.857
Total polysaccharide content (μg GE/mg)	47.310 ± 2.029

**FIGURE 3:**
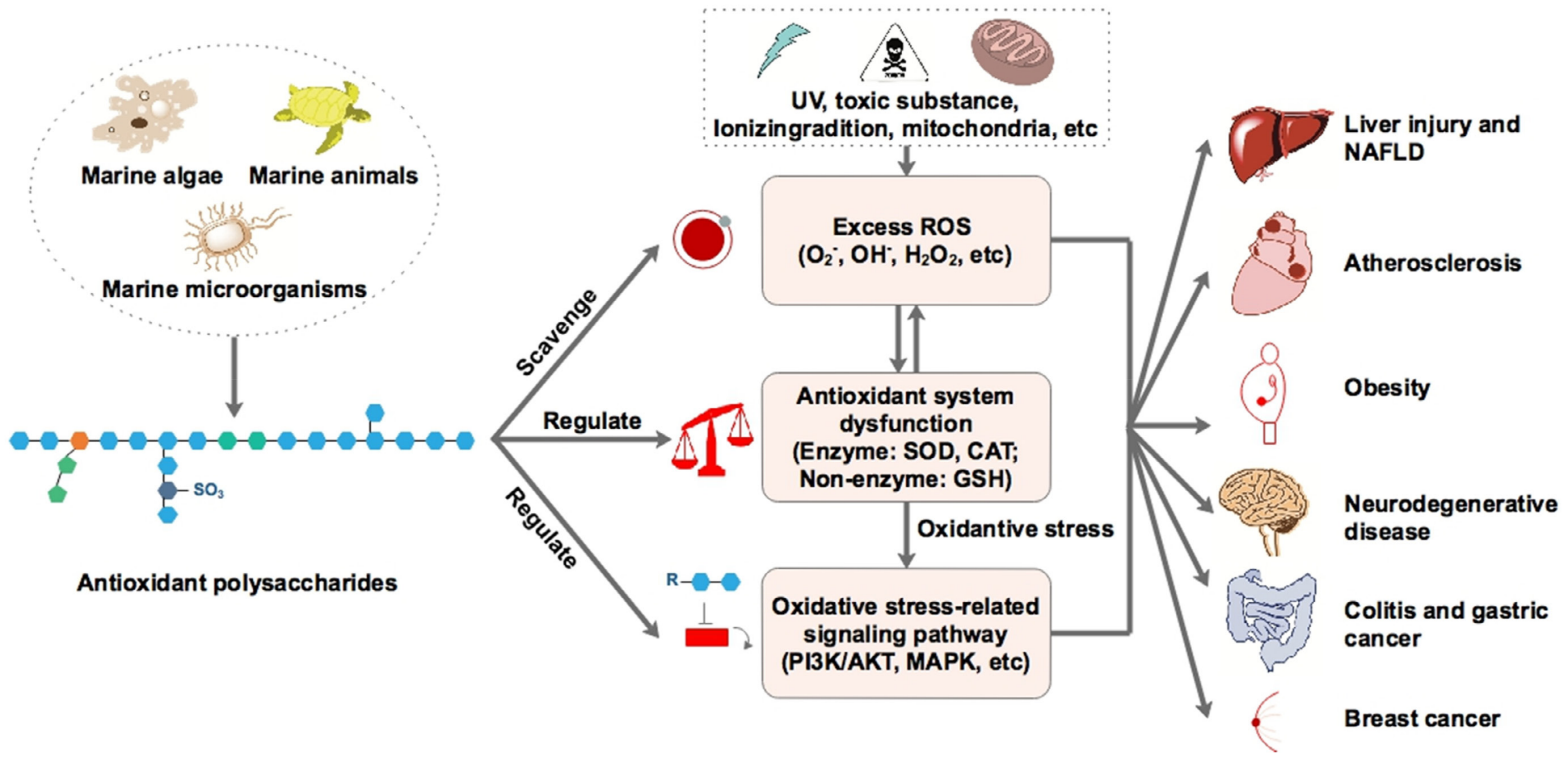
Overview of marine‐derived polysaccharides in alleviating oxidative stress‐mediated diseases (cited from Zhong et al., 2019).

Zhong et al. (2019), illustrated the antioxidant mechanisms and pharmacological effects of polysaccharides (Figure 3).

### 3.3 Identification of the chemical constituents of *Eg*H‐AE

**FIGURE 4:**
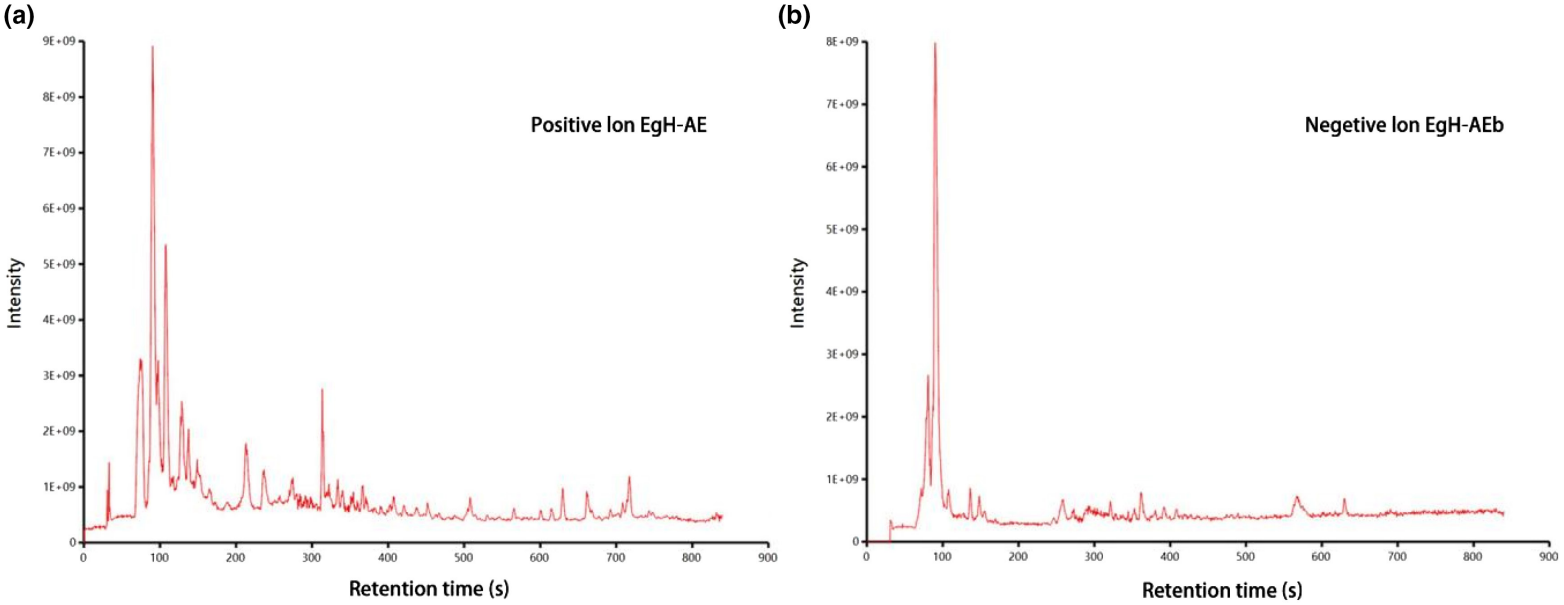
LC–MS analysis of *Eg*H‐AE. (a) Positive ion flow diagram; (b) negative ion flow diagram.

This is the first time to analyze the composition of *Eg*H‐AE. A nontargeted UPLC–MS/MS‐based method was used to analyze *Eg*H‐AE, and the obtained data then underwent bioinformatics analysis. The positive and negative ion flow diagrams of *Eg*H‐AE are shown in Figure 4. A total of 196 species of chemical constituents of *Eg*H‐AE were identified, including 111 species in positive ion mode and 85 species in negative ion mode. According to the peak response intensity of the total ion chromatogram, the 10 substances with the highest content in the *Eg*H‐AE can be preliminarily determined (Table 3). Among them, Pipecolic acid, 1,2,3‐Trihydroxybenzene, and Taurine have antioxidant activity (Aruoma et al., 1988; Sroka & Cisowski, 2003; Wang et al., 2021). Pipecolic acid, Azelaic acid, 1,2,3‐Trihydroxybenzene, and Taurine have anti‐inflammatory activity (Letavic et al., 2002; Marcinkiewicz & Kontny, 2014; Mastrofrancesco et al., 2010; Sharma et al., 2014). 5‐Aminolevulinic acid, Azelaic acid, and Taurine have cancer activity (Abo‐Zeid et al., 2018; Baliou et al., 2020; Manosroi et al., 2007). Azelaic acid has antibacterial activity (Nazzaro‐Porro, 1987). These results indicated that the *Eg*H‐AE had various biological activities. At the same time, it can also be inferred that the *Eg*H‐AE has a variety of potential effects as a functional beverage.

**TABLE 3:** LC–MS analysis of *Eg*H‐AE.

Forecast name	Formula	m/z	Retention time	Ppm	Pos/neg	Sample 1	Sample 2	Sample 3
Pipecolic acid	C_6_H_11_NO_2_	130.09	129.9	4.26	pos	17,290,152,176	17,821,126,683	17,119,511,902
5‐Aminolevulinic acid	C_5_H_9_NO_3_	132.06	237.1	22.53	pos	4,112,449,899	67,426,893.66	50,857,584.73
Azelaic acid	C_9_H_16_O_4_	187.10	302.7	6.29	neg	3,691,034,120	3,707,804,857	2,461,219,953
Geranyl diphosphate	C_10_H_20_O_7_P_2_	314.09	251.4	3.71	pos	2,993,091,621	2,999,381,346	2,487,338,137
Guanine	C_5_H_5_N_5_O	152.06	130.9	0.66	pos	2,044,394,650	2,108,126,077	2,029,668,974
Muramic acid	C_9_H_17_NO_7_	252.11	89.1	0.40	pos	1,736,443,873	1,707,489,902	1,729,676,138
2‐Dehydro‐3‐deoxy‐l‐rhamnonate	C_6_H_10_O_5_	145.05	248.7	25.05	pos	1,703,424,066	1,518,257,904	2,175,279,889
4‐Hydroxycinnamoylagmatine	C_14_H_20_N_4_O_2_	276.14	268.5	1.23	pos	1,133,579,959	807,621,617.5	1,111,552,004
1,2,3‐Trihydroxybenzene	C_6_H_6_O_3_	127.04	259.9	10.83	pos	1,089,956,426	1,340,486,189	345,604,868.5
Taurine	C_2_H_7_NO_3_S	124.04	490.3	22.08	neg	1,018,296,245	178,820,049.7	416,833,992.6

### 3.4 In vitro biocompatibility of *Eg*H‐AE

Biocompatibility, which is sometimes called “cytotoxicity”, is the most commonly used term to describe the appropriate biosafety requirements of agents or biomaterials. Their presence in bodies will not cause cell damage or affect the function of cells. Biocompatibility is also described as the ability of agents or biomaterials to have an appropriate host response in a specific application. One of the most common methods for testing biocompatibility is the MTT assay (Aoki & Saito, 2020; Mastrogiovanni et al., 2019; Stan et al., 2019).

**FIGURE 5:**
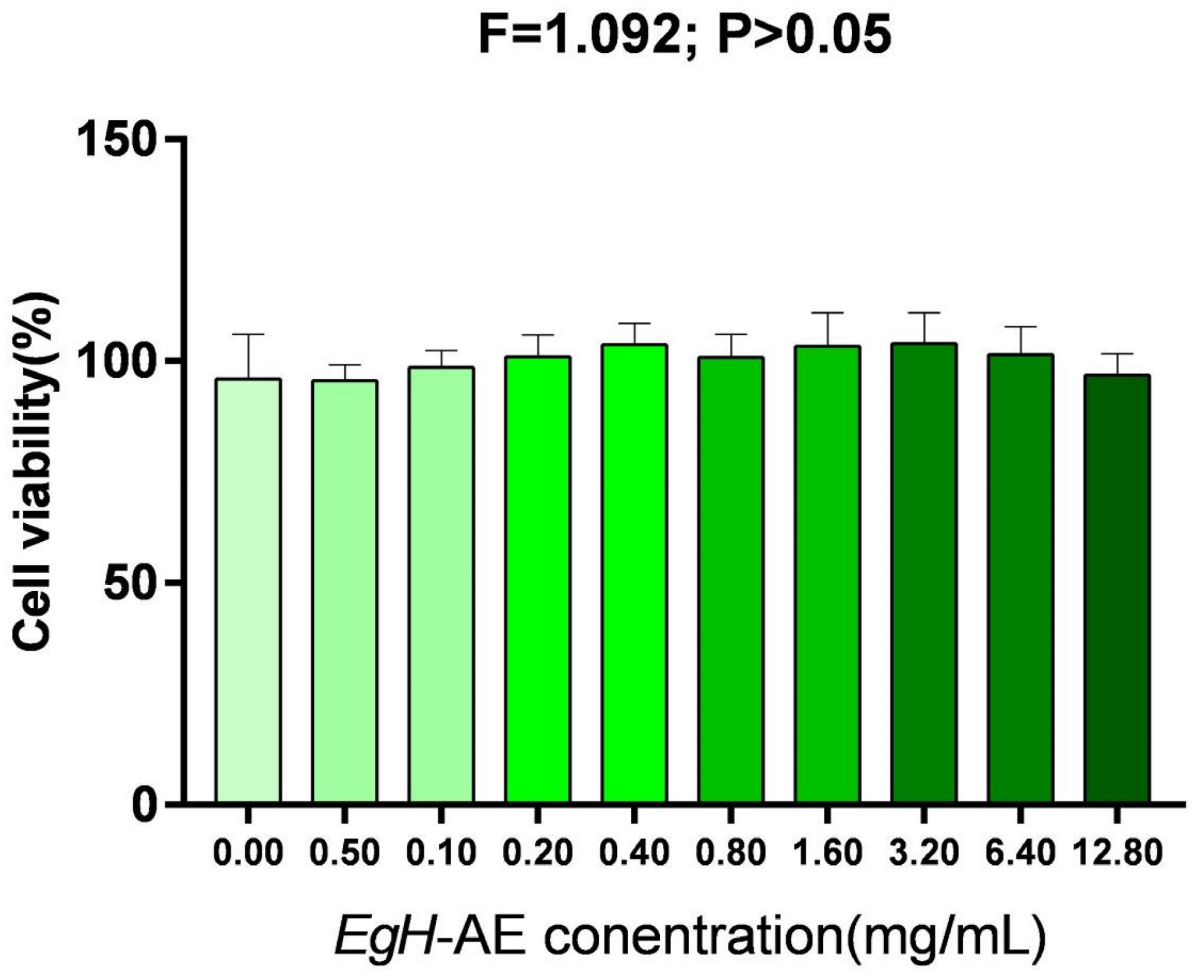
Biocompatibility of *Eg*H‐AE at different concentrations. The data were analyzed by one‐way ANOVA, *F* = 1.092 and *p* > .05, there is no statistical difference among these groups.

To assess biocompatibility, the Caco‐2 cells were treated with different concentrations of *Eg*H‐AE for 24 h. After 24 h of exposure, MTT test results (Figure 5) showed that cell viability remained statistically unchanged when compared to the untreated cells (0.00 mg/mL group). The viability of the cells incubated in the presence of the *Eg*H‐AE showed a slight change of 0.0%–10% for all of the tested doses, proving that the extract had no harmful effects on the intestinal cells.

### 3.5 Cytoprotective effect of *Eg*H‐AE against t‐BHP‐induced damage

**FIGURE 6:**
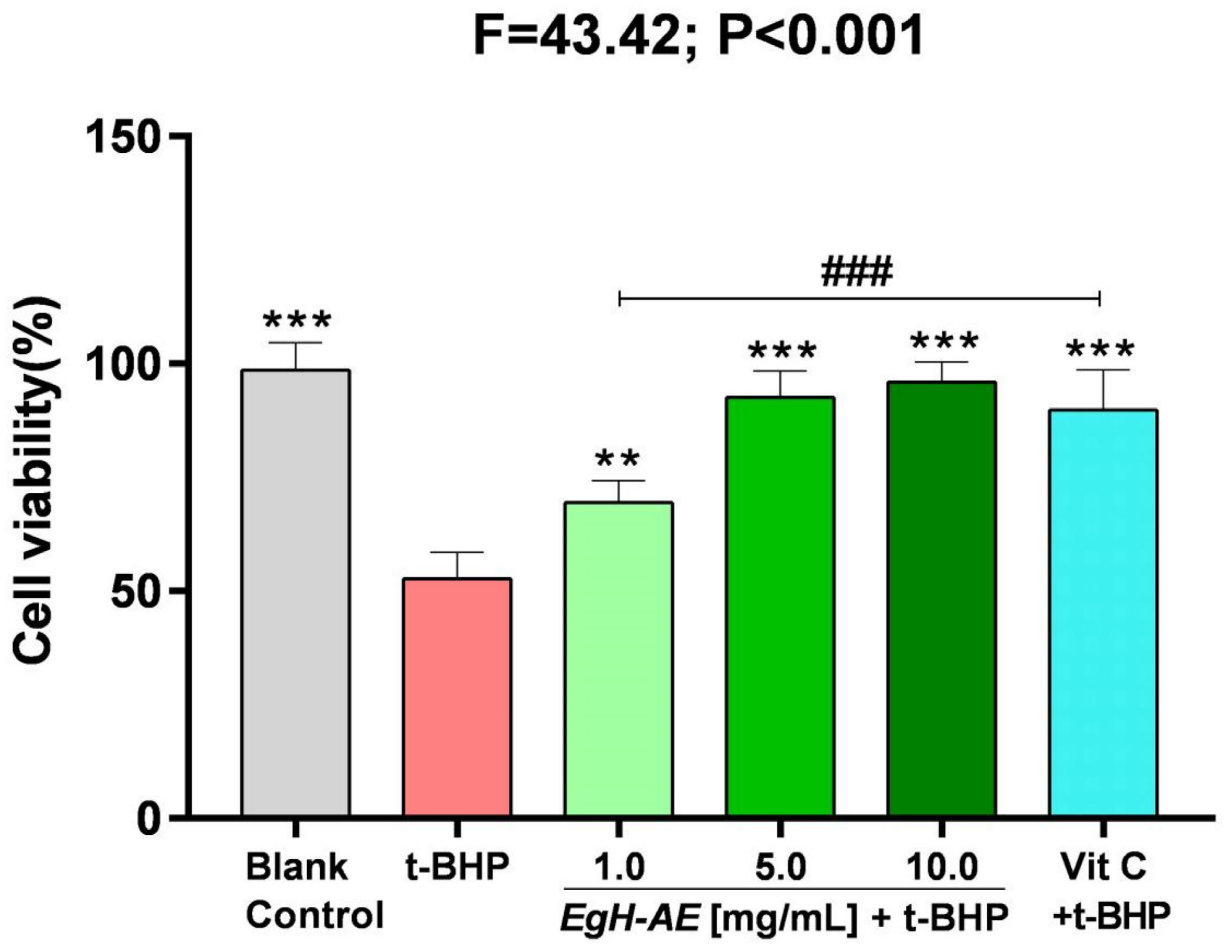
Cytoprotective effect of *Eg*H‐AE in Caco‐2 cells. The results of cellular cell viability were analyzed by one‐way ANOVA and followed by an LSD test. Asterisks indicate comparison with the t‐BHP group, *p < .05, **p < .01, ***p < .001; well numbers indicate comparison with the Vit C group, ^#^ *p* < .05, ^##^ *p* < .01, ^###^ *p* < .001.

We evaluated the cytoprotective effect of *Eg*H‐AE against oxidative stress induced with t‐BHP, by MTT assay at a concentration of 2 μmol/mL for 5 h. The results of cell viability for the different groups are shown in Figure 6.

It is clear that: (1) After 5 h, the cell viability of the cells that received the 2 μmol/mL t‐BHP treatment, decreased significantly to about 50% that of the blank control group (*p* < .001). This means that t‐BHP treatment can badly damage Caco‐2 cells; (2) Compared with the t‐BHP only group, the cell viability in the Vit C + t‐BHP group increased significantly (*p* < .001), indicating that the positive reference agent, Vit C, has a good cytoprotective effect; (3) Compared with the t‐BHP only group, the cell viability in the *Eg*H‐AE + t‐BHP groups also increased significantly (*p* < .01, *p* < .001, *p* < .001), indicating that the *Eg*H‐AE groups achieved a positive cytoprotective effect, and this effect is very closely dose‐related, a result that is similar to other studies found in the literature (Antognoni et al., 2020; Bedoya‐Ramírez et al., 2017; Dai et al., 2020).

**FIGURE 7:**
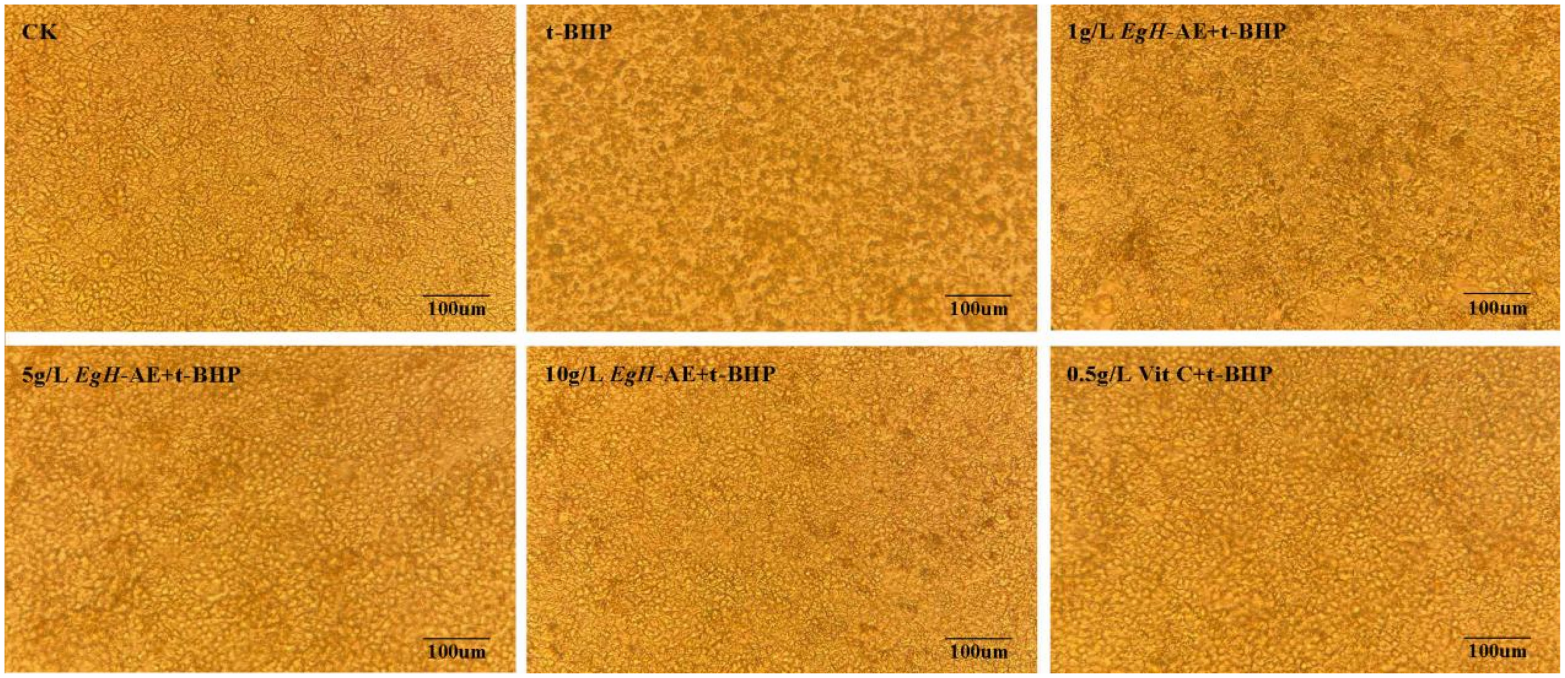
Effects of different treatments on Caco‐2 cell morphology.

The effect of different treatments on the morphology of Caco‐2 cells is shown in Figure 7. The cells of the CK group grow well, the adherent wall is firm, the cells are tightly connected in a polygonal shape, the size is uniform, the edges are distinct, and the paving stone‐like single‐layer mosaic is arranged. After 5 h of 2 μmoL/mL t‐BHP treatment, Caco‐2 cells increased the intercellular space, the morphology of the cells became blurred, and the cells began to fall off in large quantities. After the cells were preprotected by different concentrations of *Eg*H‐AE, the cell morphology was less and less affected by t‐BHP with the increase of *Eg*H‐AE treatment concentration, and the cell morphology of the 10 g/L *Eg*H‐AE + t‐BHP group was similar to that of the CK group. At the same time, after the cells were preprotected by Vit C, the effects caused by t‐BHP were primarily avoided, but the protection degree of 10 g/L *Eg*H‐AE could not be achieved.

### 3.6 Antioxidant activity of *Eg*H‐AE in Caco‐2 cells

**FIGURE 8:**
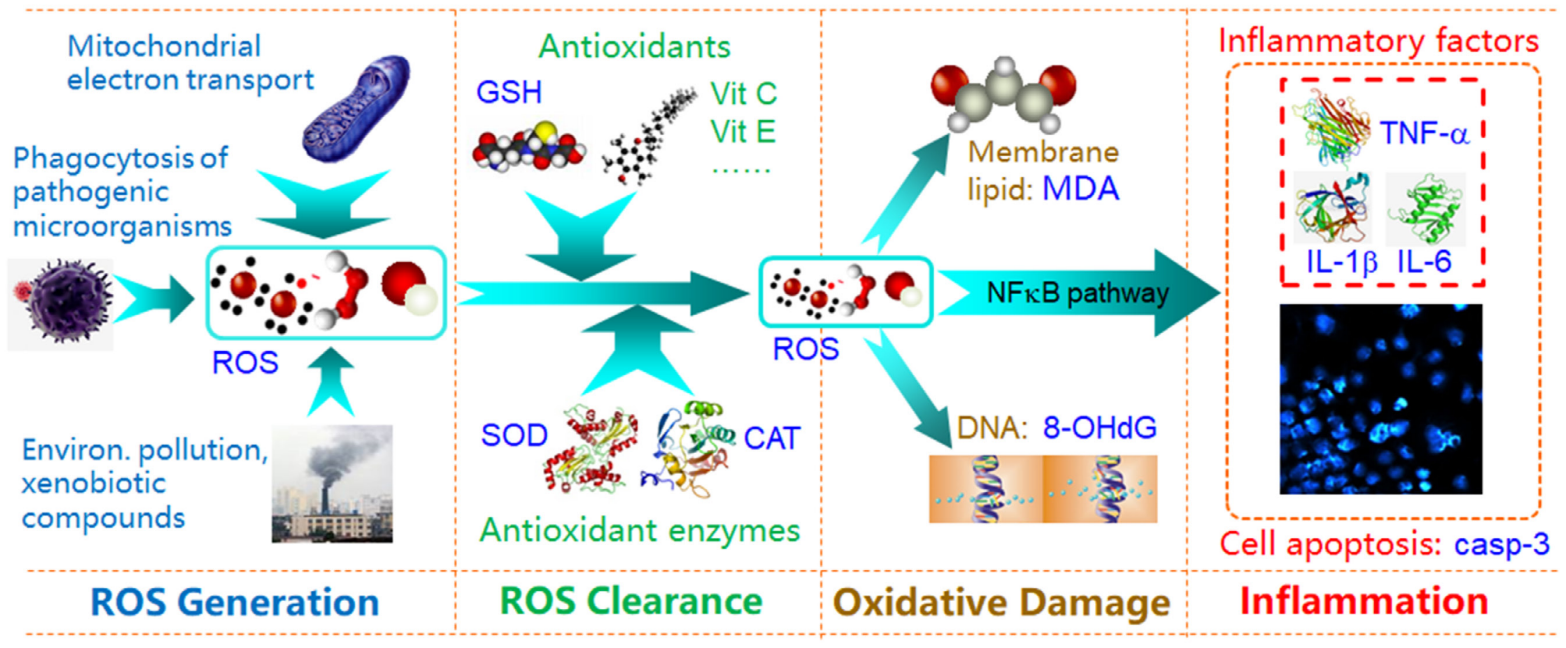
Main biomarkers indicating cellular oxidative stress and inflammation.

Figure 8 illustrates the main biomarkers for indicating cellular oxidative stress and inflammation. In this study we chose: ROS and MDA as the biomarkers for proving antioxidant activity (Bartolomei et al., 2021; Lin et al., 2020; Wang et al., 2020); TNF‐α and IL‐1β as the biomarkers for proving anti‐inflammation activity (Lameirão et al., 2020; Mastrogiovanni et al., 2019; Park et al., 2022); and 8‐OHdG as the biomarker for proving anticancer activity (Jee et al., 2020; Reuter et al., 2010; Tiwari & Mishra, 2017; Yadav et al., 2016), although it can be also be used as a biomarker of oxidative stress.

**FIGURE 9:**
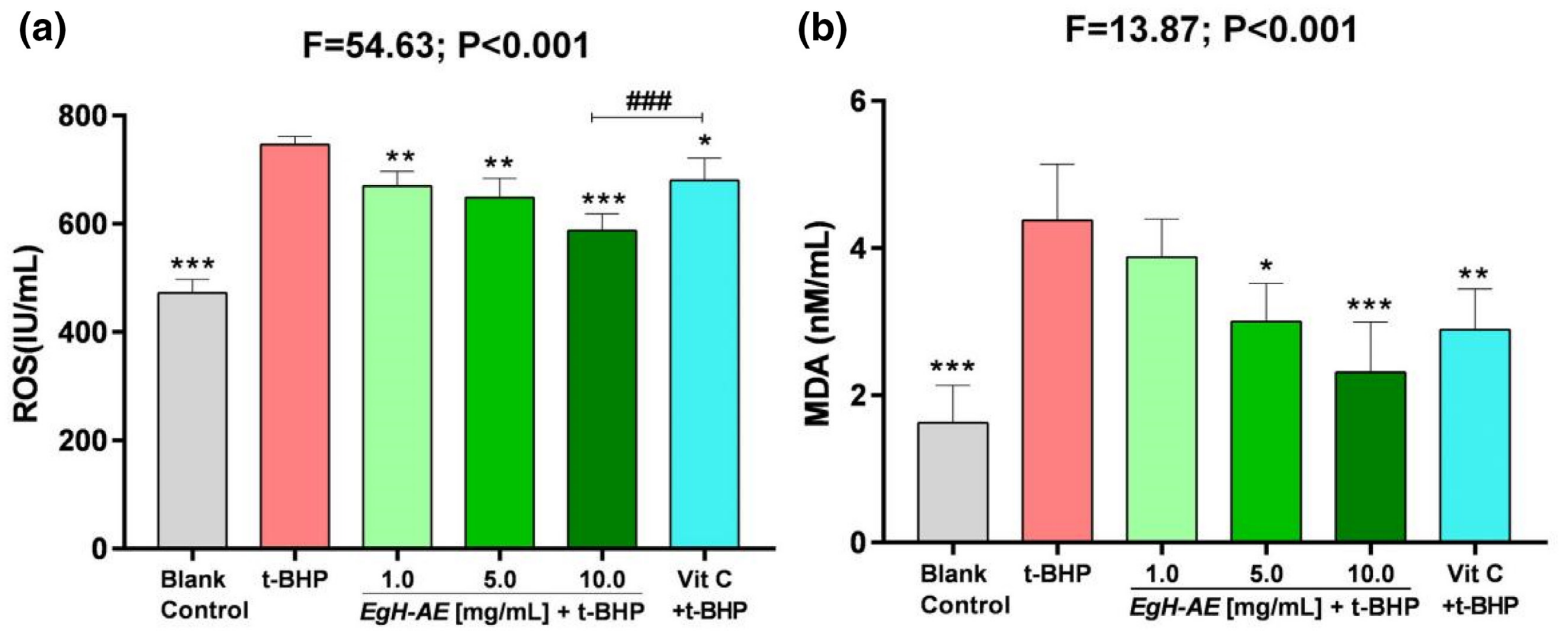
Antioxidant activity of *Eg*H‐AE in Caco‐2 cells. (a) Measured cellular ROS levels; (b) measured cellular MDA levels. The data were analyzed by one‐way ANOVA and followed by an LSD test. Asterisks indicate comparison with the t‐BHP group, *p < .05, **p < .01, ***p < .001; well numbers indicate comparison with the Vit C group, ^#^ *p* < .05, ^##^ *p* < .01, ^###^ *p* < .001.

The results of ROS determination are shown in Figure 9a: (1) The levels of ROS in Caco‐2 cells treated with 2 μmoL/mL t‐BHP, after 5 h, increased significantly in the t‐BHP group compared with the blank control group (*p* < .001). This shows that t‐BHP treatment can induce strong oxidative stress in Caco‐2 cells; (2) Compared with the t‐BHP group, the levels of ROS in the Vit C group decreased significantly (*p* < .01), indicating that the positive reference agent, Vit C, is a good antioxidant; (3) Compared with the t‐BHP group, the cellular ROS levels in each *Eg*H‐AE group decreased in a dose‐dependent manner (ns, *p* < .05, *p* < .001), indicating that *Eg*H‐AE is a very good ROS scavenger, and this scavenging effect has a good dose‐effect relationship.

Malondialdehyde levels are shown in Figure 9b: (1) The levels of MDA in Caco‐2 cells after 5 h of 2 μmoL/mL t‐BHP treatment, increased significantly in the t‐BHP group compared with the blank control group (*p* < .001). The figure shows that t‐BHP treatment can induce strong oxidative stress in Caco‐2 cells; (2) Compared with t‐BHP only group, the content of MDA in the Vit C group decreased significantly (*p* < .01), illustrating that Vit C is a good antioxidant; (3) Compared with the t‐BHP group, the cellular MDA levels in each *Eg*H‐AE group decreased in a dose‐dependent manner (ns, *p* < .05, *p* < .001). This indicates that *Eg*H‐AE has a positive effect in preventing oxidative damage of membrane lipids, and this effect has a good dose‐effect relationship.

### 3.7 Anti‐inflammatory activity of *Eg*H‐AE

**FIGURE 10:**
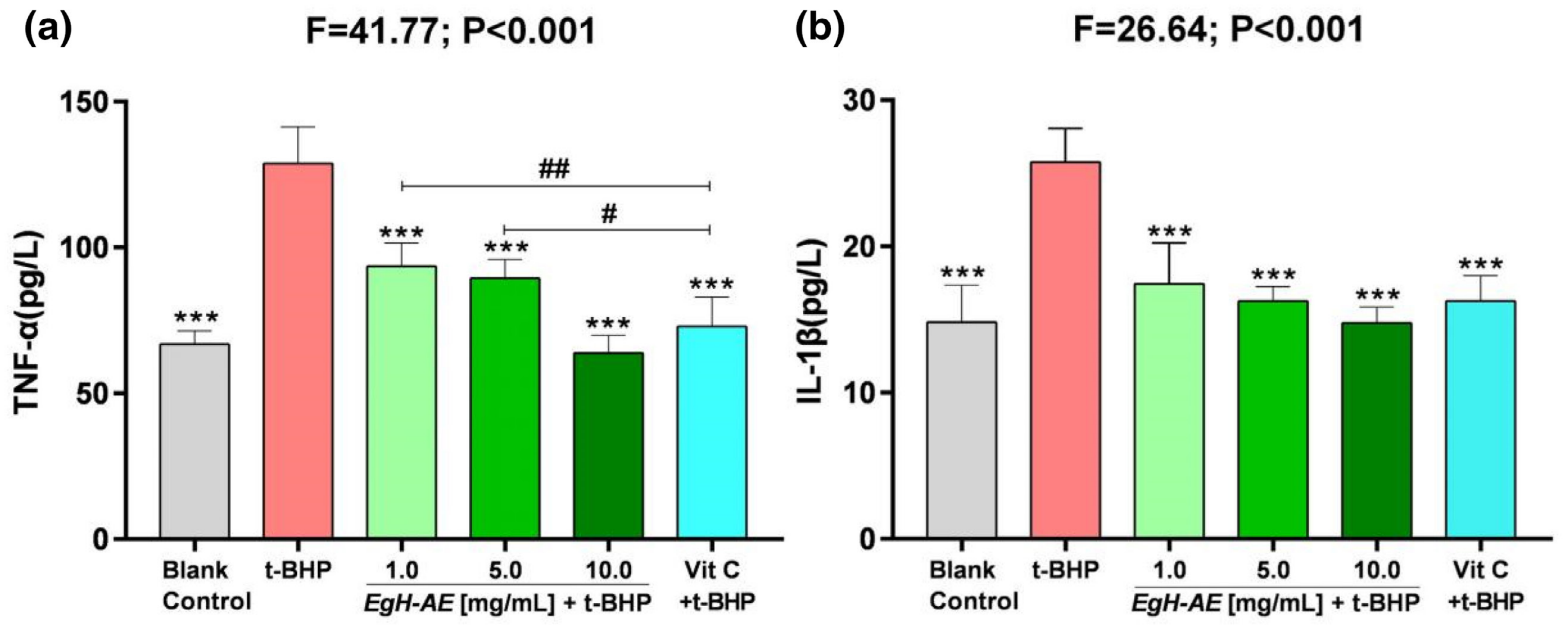
Anti‐inflammatory effect of *Eg*H‐AE in Caco‐2 Cells. (a) The levels of cellular TNF‐α; (b) the levels of cellular IL‐1β. The data were analyzed by one‐way ANOVA and followed by an LSD test. Asterisks indicate comparison with the t‐BHP group, *p < .05, **p < .01, ***p < .001; Well numbers mean comparison with the Vit C group, ^#^ *p* < .05, ^##^ *p* < .01; well numbers indicate comparison with the Vit C group, ^#^ *p* < .05, ^##^ *p* < .01, ^###^ *p* < .001.

The results for TNF‐α levels are shown in Figure 10a: (1) The levels of TNF‐α, in the Caco‐2 cells after 5 h treatment with 2 μmol/mL t‐BHP, increased significantly in the t‐BHP group compared with the blank control group (*p* < .001). It can be seen that t‐BHP treatment can induce strong inflammation in the Caco‐2 cells; (2) Compared with t‐BHP only group, levels of TNF‐α in the Vit C group decreased significantly (*p* < .01), indicating that Vit C is a very good anti‐inflammatory agent; Compared with the 1.0 and 5.0 *Eg*H‐AE groups, Vit C has better anti‐inflammation activity (*p* < .01 and *p* < .05); (3) Compared with the t‐BHP group, levels of cellular TNF‐α in each of the *Eg*H‐AE groups decreased in a dose‐dependent manner (*p* < .001, *p* < .001, *p* < .001), indicating that *Eg*H‐AE is a very good anti‐inflammatory agent, and this effect also has a dose‐effect relationship.

IL‐1β levels are shown in Figure 10b: (1) The levels of IL‐1β, in the Caco‐2 cells after 5 h of treatment with 2 μmoL/mL t‐BHP, increased significantly in the t‐BHP group compared with the blank control group (*p* < .001). The figure shows that t‐BHP treatment can induce strong oxidative stress in the Caco‐2 cells; (2) Compared with the t‐BHP group, the levels of IL‐1β in the Vit C group decreased significantly (*p* < .01), indicating that Vit C is a good antioxidant; (3) Compared with the t‐BHP group, the levels of cellular IL‐1β in each *Eg*H‐AE group decreased gradually (*p* < .001, *p* < .001, *p* < .001), indicating that *Eg*H‐AE is a very good anti‐inflammatory agent, and this effect also has a dose‐effect relationship.

### 3.8 Anticancer activities of *Eg*H‐AE

8‐OHdG is a special biomarker, because it is one of the endpoints of oxidative stress, but is also an initiator of carcinogenesis (Reuter et al., 2010). The ROS‐derived DNA damage includes the generation of 8‐hydroxyguanosine, the hydrolysis product of which is 8‐hydroxydeoxyguanosine (8‐OHdG). 8‐OHdG is the most widely used indicator of a radical attack on DNA (Marnett, 2000; Wiseman & Halliwell, 1996). 8‐OHdG is strongly implicated in carcinogenesis progression. For example, in breast carcinomas, levels of 8‐OHdG have been reported to be 8–17‐fold higher in primary breast tumors compared with healthy breast tissue (Musarrat et al., 1996; Valavanidis et al., 2009).

**FIGURE 11:**
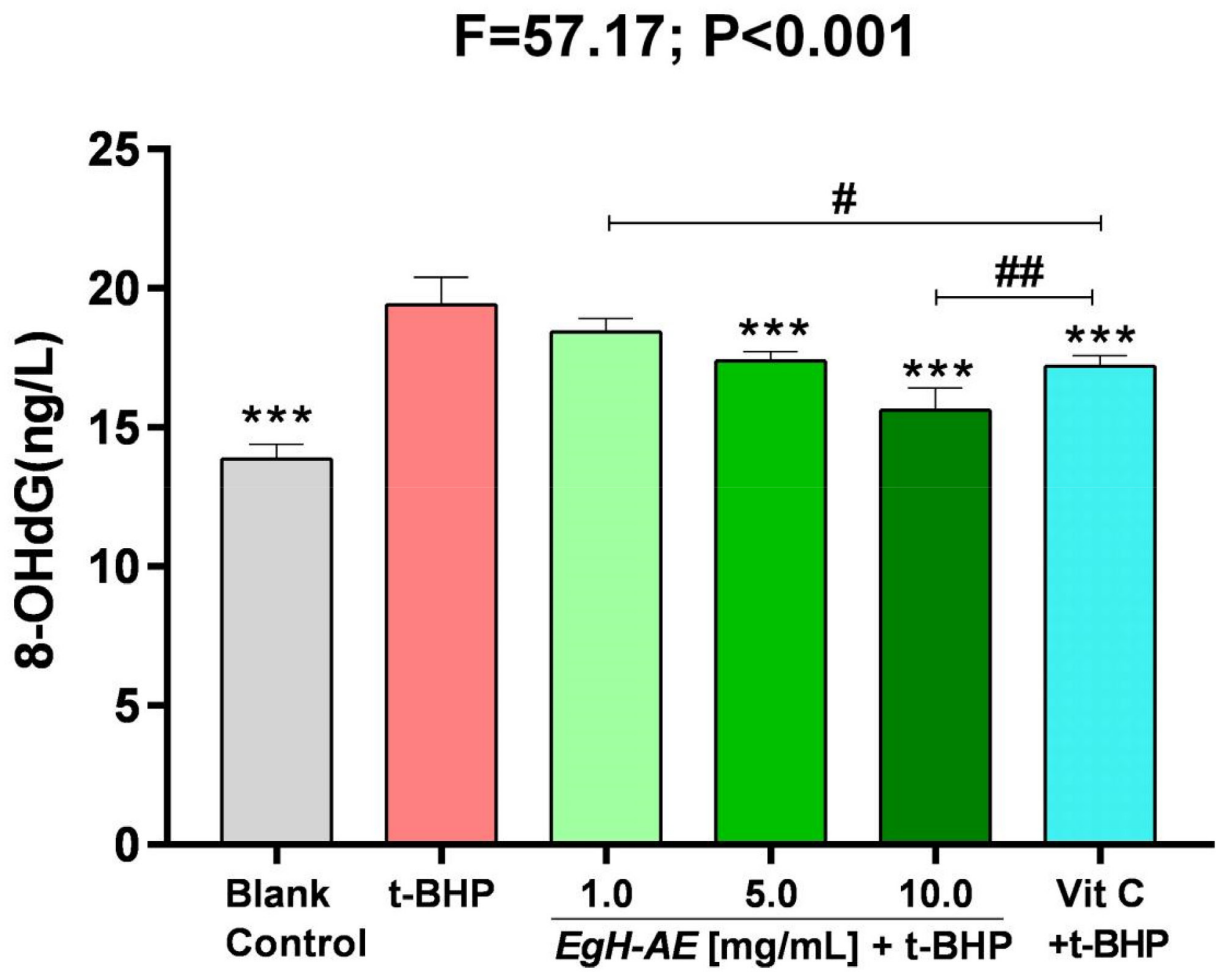
Anticancer activity of *Eg*H‐AE in Caco‐2 cells. The results of cellular 8‐OHdG were analyzed by one‐way ANOVA and followed by an LSD test. Asterisks indicate comparison with the t‐BHP group, *p < .05, **p < .01, ***p < .001; well numbers indicate comparison with the Vit C group, ^#^ *p* < .05, ^##^ *p* < .01, ^###^ *p* < .001.

The results of 8‐OHdG determination are shown in Figure 11: (1) The levels of 8‐OHdG, in Caco‐2 cells after 5 h of treatment with 2 μmoL/mL t‐BHP increased significantly, in the t‐BHP group compared with the blank control group (*p* < .001). This means that t‐BHP can cause extensive DNA oxidative damage, which may then induce DNA base mismatch and sequence point mutation in Caco‐2 cells; (2) Compared with the t‐BHP group, levels of 8‐OHdG in the Vit C group decreased significantly (*p* < .001), indicating that Vit C is protective against cancer; Compared with the 1.0 *Eg*H‐AE group, Vit C is a better anticancer agent (*p* < .05), but compared with the 10.0 *Eg*H‐AE group, Vit C is not as effective an anticancer agent (*p* < .01); (3) Compared with the t‐BHP group, the levels of cellular 8‐OHdG in each *Eg*H‐AE group decreased in a dose‐dependent manner (ns, *p* < .001, *p* < .001), indicating that a higher dose of *Eg*H‐AE has very good anticancer properties, and this anticancer effect also has a dose‐effect relationship.

## 4 CONCLUSION

In this study, we found that *E. goetzei* Henn aqueous extract (*Eg*H‐AE) is very biocompatible with the Caco‐2 cell line, and has good cytoprotective, antioxidant, anti‐inflammatory, and anticancer properties. The results of this study support the idea that *Eg*H‐AE, the historical herbal tea used in Yunnan province of China, may be used to develop a functional beverage that can be given to people with a high BMI to protect against obesity‐induced diseases.

## AUTHOR CONTRIBUTIONS

Y.X., X.W., Y.X‐Y. and S.R. conceptualized the study; S.R., N.J., and L.L‐J. involved in methodology; N.J. provided the software; Y.X‐Y. and S.R. involved in validation; N.J. analyzed the data formally; N.J., Z.C‐F., C.Y‐T., and G.Y‐T involved in investigation; X.W. provided the resources; Y.Y‐X. and S.R. involved in data curation; N.J. wrote the original draft preparation; Y.X‐Y. and S.R. involved in review and editing; N.J. and Y.X involved in visualization process; X.W. involved in supervision, project administration, and funding acquisition. All authors have read and agreed to the published version of the manuscript.

## FUNDING INFORMATION

This research was funded by “Development of diagnostic instrument for oxidative inflammation: determination of blood ROS, MDA and urine 8‐OHdG”, grant number “202101BA070001‐115”.

## CONFLICT OF INTEREST STATEMENT

The authors declare that they have no known competing financial interests or personal relationships that could have appeared to influence the work reported in this paper.

## ETHICS STATEMENT

This research did not include biological agents of risk, or clinical trials with humans or animal experiments.

## Data Availability

Data used for this study are available on request through the corresponding author, although all the relevant data have been provided here.

## References

[fsn33335-bib-0001] Abbasi‐Parizad, P. , De Nisi, P. , Adani, F. , Pepé Sciarria, T. , Squillace, P. , Scarafoni, A. , Iametti, A. , & Scaglia, B. (2020). Antioxidant and anti‐inflammatory activities of the crude extracts of raw and fermented tomato pomace and their correlations with aglycate‐polyphenols. Antioxidants, 9(2), 179. 10.3390/antiox9020179 32098217 PMC7070286

[fsn33335-bib-0002] Abo‐Zeid, M. A. , Abo‐Elfadl, M. T. , & Mostafa, S. M. (2018). Photodynamic therapy using 5‐aminolevulinic acid triggered DNA damage of adenocarcinoma breast cancer and hepatocellular carcinoma cell lines. Photodiagnosis and Photodynamic Therapy, 21, 351–356. 10.1016/j.pdpdt.2018.01.011 29355735

[fsn33335-bib-0003] Ainsworth, E. A. , & Gillespie, K. M. (2007). Estimation of total phenolic content and other oxidation substrates in plant tissues using Folin–Ciocalteu reagent. Nature Protocols, 2(4), 875–877. 10.1038/nprot.2007.102 17446889

[fsn33335-bib-0004] Annett, S. , Moore, G. , & Robson, T. (2020). Obesity and cancer metastasis: Molecular and translational perspectives. Cancers, 12(12), 3798. 10.3390/cancers12123798 33339340 PMC7766668

[fsn33335-bib-0005] Antognoni, F. , Potente, G. , Mandrioli, R. , Angeloni, C. , Freschi, M. , Malaguti, M. , Hrelia, S. , Lugli, S. , Gennari, F. , Muzzi, E. , & Tartarini, S. (2020). Fruit quality characterization of new sweet cherry cultivars as a good source of bioactive phenolic compounds with antioxidant and neuroprotective potential. Antioxidants, 9(8), 677. 10.3390/antiox9080677 32731644 PMC7463759

[fsn33335-bib-0006] Aoki, K. , & Saito, N. (2020). Biocompatibility and carcinogenicity of carbon nanotubes as biomaterials. Nanomaterials, 10(2), 264. 10.3390/nano10020264 32033249 PMC7075247

[fsn33335-bib-0007] Aruoma, O. I. , Halliwell, B. , Hoey, B. M. , & Butler, J. (1988). The antioxidant action of taurine, hypotaurine and their metabolic precursors. Biochemical Journal, 256(1), 251–255. 10.1042/bj2560251 2851980 PMC1135395

[fsn33335-bib-0008] Baliou, S. , Kyriakopoulos, A. M. , Spandidos, D. A. , & Zoumpourlis, V. (2020). Role of taurine, its haloamines and its lncRNA TUG1 in both inflammation and cancer progression. On the road to therapeutics? International Journal of Oncology, 57(3), 631–664. 10.3892/ijo.2020.5100 32705269 PMC7384849

[fsn33335-bib-0009] Bartolomei, M. , Bollati, C. , Bellumori, M. , Cecchi, L. , Cruz‐Chamorro, I. , Santos‐Sánchez, G. , Ranaldi, G. , Ferruzza, S. , Sambuy, Y. , Arnoldi, A. , Mulinacci, N. , & Lammi, C. (2021). Extra virgin olive oil phenolic extract on human hepatic HepG2 and intestinal Caco‐2 cells: Assessment of the antioxidant activity and intestinal trans‐epithelial transport. Antioxidants, 10(1), 118. 10.3390/antiox10010118 33467632 PMC7829860

[fsn33335-bib-0010] Battineni, G. , Sagaro, G. G. , Chintalapudi, N. , Amenta, F. , Tomassoni, D. , & Tayebati, S. K. (2021). Impact of obesity‐induced inflammation on cardiovascular diseases (CVD). International Journal of Molecular Sciences, 22(9), 4798. 10.3390/ijms22094798 33946540 PMC8125716

[fsn33335-bib-0011] Bedoya‐Ramírez, D. , Cilla, A. , Contreras‐Calderón, J. , & Alegría‐Torán, A. (2017). Evaluation of the antioxidant capacity, furan compounds and cytoprotective/cytotoxic effects upon Caco‐2 cells of commercial colombian coffee. Food Chemistry, 219, 364–372. 10.1016/j.foodchem.2016.09.159 27765239

[fsn33335-bib-0012] Brand‐Williams, W. , Cuvelier, M. E. , & Berset, C. (1995). Use of a free radical method to evaluate antioxidant activity. LWT – Food Science and Technology, 28(1), 25–30. 10.1016/S0023-6438(95)80008-5

[fsn33335-bib-0013] Brewer, C. J. , & Balen, A. H. (2010). Focus on obesity. Reproduction, 140(3), 347–364. 10.1530/REP-09-0568 20395425

[fsn33335-bib-0014] Corsetto, P. A. , Montorfano, G. , Zava, S. , Colombo, I. , Ingadottir, B. , Jonsdottir, R. , Sveinsdottir, K. , & Rizzo, A. M. (2020). Characterization of antioxidant potential of seaweed extracts for enrichment of convenience food. Antioxidants, 9(3), 249. 10.3390/antiox9030249 32204441 PMC7139466

[fsn33335-bib-0015] Dai, M. , Li, C. , Yang, Z. , Sui, Z. , Li, J. , Dong, P. , & Liang, X. (2020). The astaxanthin aggregation pattern greatly influences its antioxidant activity: A comparative study in Caco‐2 cells. Antioxidants, 9(2), 126. 10.3390/antiox9020126 32024215 PMC7070916

[fsn33335-bib-0016] Deng, W. , Liu, K. , Cao, S. , Sun, J. , Zhong, B. , & Chun, J. (2020). Chemical composition, antimicrobial, antioxidant, and antiproliferative properties of grapefruit essential oil prepared by molecular distillation. Molecules, 25(1), 217. 10.3390/molecules25010217 31948058 PMC6982870

[fsn33335-bib-0017] Dong, Y. , Hou, Q. , Sun, M. , Sun, J. , & Zhang, B. (2020). Targeted isolation of antioxidant constituents from *Plantago asiatica* L. and in vitro activity assay. Molecules, 25(8), 1825. 10.3390/molecules25081825 32316264 PMC7221530

[fsn33335-bib-0018] Dubois, M. , Gilles, K. A. , Hamilton, J. K. , Rebers, P. A. , & Smith, F. A. J. N. (1951). A colorimetric method for the determination of sugars. Nature, 168(4265), 167. 10.1038/168167a0 14875032

[fsn33335-bib-0019] Ferhi, S. , Santaniello, S. , Zerizer, S. , Cruciani, S. , Fadda, A. , Sanna, D. , Dore, A. , Maioli, M. , & D'hallewin, G. (2019). Total phenols from grape leaves counteract cell proliferation and modulate apoptosis‐related gene expression in MCF‐7 and HepG2 human cancer cell lines. Molecules, 24(3), 612. 10.3390/molecules24030612 30744145 PMC6384979

[fsn33335-bib-0020] Gonçalves, A. C. , Rodrigues, M. , Santos, A. O. , Alves, G. , & Silva, L. R. (2018). Antioxidant status, antidiabetic properties and effects on Caco‐2 cells of colored and non‐colored enriched extracts of sweet cherry fruits. Nutrients, 10(11), 1688. 10.3390/nu10111688 30400658 PMC6266284

[fsn33335-bib-0021] Jee, S. C. , Kim, M. , Kim, K. S. , Kim, H. S. , & Sung, J. S. (2020). Protective effects of myricetin on benzo[a]pyrene‐induced 8‐hydroxy‐2′‐deoxyguanosine and BPDE‐DNA adduct. Antioxidants, 9(5), 446. 10.3390/antiox9050446 32455619 PMC7278665

[fsn33335-bib-0022] Jikai, L. , Jianwen, T. , Zejun, D. , Zhihui, D. , Xianghua, W. , & Peigui, L. (2002). Neoengleromycin, a novel compound from *Engleromyces goetzii* . Helvetica Chimica Acta, 85(5), 1439–1442. 10.1002/1522-2675(200205)85:5<1439::AID-HLCA1439>3.0.CO;2-X

[fsn33335-bib-0023] Kern, L. , Mittenbühler, M. J. , Vesting, A. J. , Ostermann, A. L. , Wunderlich, C. M. , & Wunderlich, F. T. (2018). Obesity‐induced TNFα and IL‐6 signaling: The missing link between obesity and inflammation—Driven liver and colorectal cancers. Cancers, 11(1), 24. 10.3390/cancers11010024 30591653 PMC6356226

[fsn33335-bib-0024] Kim, J. , & Lee, J. (2021). Role of obesity‐induced inflammation in the development of insulin resistance and type 2 diabetes: History of the research and remaining questions. Annals of Pediatric Endocrinology & Metabolism, 26(1), 1–13. 10.6065/apem.2040188.094 33819954 PMC8026341

[fsn33335-bib-0025] Kwaifa, I. K. , Bahari, H. , Yong, Y. K. , & Noor, S. M. (2020). Endothelial dysfunction in obesity‐induced inflammation: Molecular mechanisms and clinical implications. Biomolecules, 10(2), 291. 10.3390/biom10020291 32069832 PMC7072669

[fsn33335-bib-0026] Lameirão, F. , Pinto, D. , Vieira, E. F. , Peixoto, A. F. , Freire, C. , Sut, S. , Dall'Acqua, S. , Costa, P. , Delerue‐Matos, C. , & Rodrigues, F. (2020). Green‐sustainable recovery of phenolic and antioxidant compounds from industrial chestnut shells using ultrasound‐assisted extraction: Optimization and evaluation of biological activities in vitro. Antioxidants, 9(3), 267. 10.3390/antiox9030267 32213812 PMC7139998

[fsn33335-bib-0027] Land Lail, H. , Feresin, R. G. , Hicks, D. , Stone, B. , Price, E. , & Wanders, D. (2021). Berries as a treatment for obesity‐induced inflammation: Evidence from preclinical models. Nutrients, 13(2), 334. 10.3390/nu13020334 33498671 PMC7912458

[fsn33335-bib-0028] Letavic, M. A. , Axt, M. Z. , Barberia, J. T. , Carty, T. J. , Danley, D. E. , Geoghegan, K. F. , Halim, N. S. , Hoth, L. R. , Kamath, A. V. , Laird, E. R. , Lopresti‐Morrow, L. L. , McClure, K. F. , Mitchell, P. G. , Natarajan, V. , Nor, M. C. , Padit, J. , Reeves, L. , Schulte, G. K. , Snow, S. L. , … Chul, H. Y. (2002). Synthesis and biological activity of selective pipecolic acid‐based TNF‐α converting enzyme (TACE) inhibitors. Bioorganic & Medicinal Chemistry Letters, 12(10), 1387–1390. 10.1016/S0960-894X(02)00183-X 11992783

[fsn33335-bib-0029] Lin, H. Y. , Weng, S. W. , Shen, F. C. , Chang, Y. H. , Lian, W. S. , Hsieh, C. H. , Chuang, J. H. , Lin, T. K. , Liou, C. W. , Chang, S. C. , Lin, C. Y. , & Su, Y. J. (2019). Abrogation of toll‐like receptor 4 mitigates obesity‐induced oxidative stress, proinflammation, and insulin resistance through metabolic reprogramming of mitochondria in adipose tissue. Antioxidants & Redox Signaling, 33(2), 66–86. 10.1089/ars.2019.7737 31950846

[fsn33335-bib-0030] Lin, X. , Liu, K. , Yin, S. , Qin, Y. , Shen, P. , & Peng, Q. (2020). A novel pectic polysaccharide of jujube pomace: Structural analysis and intracellular antioxidant activities. Antioxidants, 9, 127. 10.3390/antiox9020127 32024245 PMC7070808

[fsn33335-bib-0031] Lv, J. M. , Gouda, M. , Zhu, Y. Y. , Ye, X. Q. , & Chen, J. C. (2021). Ultrasound‐assisted extraction optimization of proanthocyanidins from kiwi (*Actinidia chinensis*) leaves and evaluation of its antioxidant activity. Antioxidants, 10(8), 1317. 10.3390/antiox10081317 34439565 PMC8389255

[fsn33335-bib-0032] Manosroi, A. , Panyosak, A. , Rojanasakul, Y. , & Manosroi, J. (2007). Characteristics and anti‐proliferative activity of azelaic acid and its derivatives entrapped in bilayer vesicles in cancer cell lines. Journal of Drug Targeting, 15(5), 334–341. 10.1080/10611860701349315 17541842

[fsn33335-bib-0033] Marcinkiewicz, J. , & Kontny, E. (2014). Taurine and inflammatory diseases. Amino Acids, 46(1), 7–20. 10.1007/s00726-012-1361-4 22810731 PMC3894431

[fsn33335-bib-0034] Marnett, L. J. (2000). Oxyradicals and DNA damage. Carcinogenesis, 21(3), 361–370. 10.1093/carcin/21.3.361 10688856

[fsn33335-bib-0035] Mastrofrancesco, A. , Ottaviani, M. , Aspite, N. , Cardinali, G. , Izzo, E. , Graupe, K. , Zouboulis, C. C. , Camera, E. , & Picardo, M. (2010). Azelaic acid modulates the inflammatory response in normal human keratinocytes through PPARγ activation. Experimental Dermatology, 19(9), 813–820. 10.1111/j.1600-0625.2010.01107.x 20545756

[fsn33335-bib-0036] Mastrogiovanni, F. , Mukhopadhya, A. , Lacetera, N. , Ryan, M. T. , Romani, A. , Bernini, R. , & Sweeney, T. (2019). Anti‐inflammatory effects of pomegranate peel extracts on in vitro human intestinal Caco‐2 cells and ex vivo porcine colonic tissue explants. Nutrients, 11(3), 548. 10.3390/nu11030548 30841512 PMC6471410

[fsn33335-bib-0037] Miller, N. J. , Rice‐Evans, C. , Davies, M. J. , Gopinathan, V. , & Milner, A. (1993). A novel method for measuring antioxidant capacity and its application to monitoring the antioxidant status in premature neonates. Clinical Science, 84(4), 407–412. 10.1042/cs0840407 8482045

[fsn33335-bib-0038] Mosmann, T. (1983). Rapid colorimetric assay for cellular growth and survival: Application to proliferation and cytotoxicity assays. Journal of Immunological Methods, 65(1–2), 55–63. 10.1016/0022-1759(83)90303-4 6606682

[fsn33335-bib-0039] Musarrat, J. , Arezina‐Wilson, J. , & Wani, A. A. (1996). Prognostic and aetiological relevance of 8‐hydroxyguanosine in human breast carcinogenesis. European Journal of Cancer, 32(7), 1209–1214. 10.1016/0959-8049(96)00031-7 8758255

[fsn33335-bib-0040] Mwangi, R. W. , Macharia, J. M. , Wagara, I. N. , & Bence, R. L. (2022). The antioxidant potential of different edible and medicinal mushrooms. Biomedicine & Pharmacotherapy, 147, 112621. 10.1016/j.biopha.2022.112621 35026489

[fsn33335-bib-0041] Navarro, M. , Arnaez, E. , Moreira, I. , Quesada, S. , Azofeifa, G. , Wilhelm, K. , Vargas, F. , & Chen, P. (2019). Polyphenolic characterization, antioxidant, and cytotoxic activities of *Mangifera indica* cultivars from Costa Rica. Food, 8(9), 384. 10.3390/foods8090384 PMC6769667 31480721

[fsn33335-bib-0042] Nazzaro‐Porro, M. (1987). Azelaic acid. Journal of the American Academy of Dermatology, 17(6), 1033–1041. 10.1016/S0190-9622(87)70294-1 2963038

[fsn33335-bib-0043] Ostrand‐Rosenberg, S. (2021). Myeloid‐derived suppressor cells: Facilitators of cancer and obesity‐induced cancer. Annual Review of Cancer Biology, 5, 17–38. 10.1146/annurev-cancerbio-042120-105240

[fsn33335-bib-0044] Pan, X. Y. , Wang, Y. M. , Li, L. , Chi, C. F. , & Wang, B. (2019). Four antioxidant peptides from protein hydrolysate of red stingray (*Dasyatis akajei*) cartilages: Isolation, identification, and in vitro activity evaluation. Marine Drugs, 17(5), 263. 10.3390/md17050263 31058809 PMC6562685

[fsn33335-bib-0045] Park, Y. J. , Yang, H. J. , Li, W. , Oh, Y. C. , & Go, Y. (2022). *Menthae herba* attenuates neuroinflammation by regulating CREB/Nrf2/HO‐1 pathway in BV2 microglial cells. Antioxidants, 11(4), 649. 10.3390/antiox11040649 35453334 PMC9029636

[fsn33335-bib-0046] Rajesh, Y. , & Sarkar, D. (2021). Association of adipose tissue and adipokines with development of obesity‐induced liver cancer. International Journal of Molecular Sciences, 22(4), 2163. 10.3390/ijms22042163 33671547 PMC7926723

[fsn33335-bib-0047] Ramarathnam, N. , Osawa, T. , Ochi, H. , & Kawakishi, S. (1995). The contribution of plant food antioxidants to human health. Trends in Food Science & Technology, 6(3), 75–82. 10.1016/S0924-2244(00)88967-0

[fsn33335-bib-0048] Ren, D. , Jiao, Y. , Yang, X. , Yuan, L. , Guo, J. , & Zhao, Y. (2015). Antioxidant and antitumor effects of polysaccharides from the fungus *Pleurotus abalonus* . Chemico‐Biological Interactions, 237, 166–174. 10.1016/j.cbi.2015.06.017 26091901

[fsn33335-bib-0049] Reuter, S. , Gupta, S. C. , Chaturvedi, M. M. , & Aggarwal, B. B. (2010). Oxidative stress, inflammation, and cancer: How are they linked? Free Radical Biology and Medicine, 49(11), 1603–1616. 10.1016/j.freeradbiomed.2010.09.006 20840865 PMC2990475

[fsn33335-bib-0050] Sharma, N. , Samarakoon, K. W. , Gyawali, R. , Park, Y. H. , Lee, S. J. , Oh, S. J. , Lee, T. H. , & Jeong, D. K. (2014). Evaluation of the antioxidant, anti‐inflammatory, and anticancer activities of *Euphorbia hirta* ethanolic extract. Molecules, 19(9), 14567–14581. 10.3390/molecules190914567 25225720 PMC6271915

[fsn33335-bib-0051] Shen, H. H. , Peterson, S. J. , Bellner, L. , Choudhary, A. , Levy, L. , Gancz, L. , Sasson, A. , Trainer, J. , Rezzani, R. , Resnick, A. , Stec, D. E. , & Abraham, N. G. (2020). Cold‐pressed *Nigella sativa* oil standardized to 3% thymoquinone potentiates omega‐3 protection against obesity‐induced oxidative stress, inflammation, and markers of insulin resistance accompanied with conversion of white to beige fat in mice. Antioxidants, 9(6), 489. 10.3390/antiox9060489 32512788 PMC7346210

[fsn33335-bib-0052] Spizzirri, U. G. , Caputo, P. , Oliviero Rossi, C. , Crupi, P. , Muraglia, M. , Rago, V. , Malivindi, R. , Clodoveo, M. L. , Restuccia, D. , & Aiello, F. (2022). A tara gum/olive mill wastewaters phytochemicals conjugate as a new ingredient for the formulation of an antioxidant‐enriched pudding. Food, 11(2), 158. 10.3390/foods11020158 PMC8774902 35053891

[fsn33335-bib-0053] Sroka, Z. , & Cisowski, W. (2003). Hydrogen peroxide scavenging, antioxidant and anti‐radical activity of some phenolic acids. Food and Chemical Toxicology, 41(6), 753–758. 10.1016/S0278-6915(02)00329-0 12738180

[fsn33335-bib-0054] Stan, M. S. , Voicu, S. N. , Caruntu, S. , Nica, I. C. , Olah, N. K. , Burtescu, R. , Balta, C. , Rosu, M. , Herman, H. , Hermenean, A. , & Dinischiotu, A. (2019). Antioxidant and anti‐inflammatory properties of a *Thuja occidentalis* mother tincture for the treatment of ulcerative colitis. Antioxidants, 8(9), 416. 10.3390/antiox8090416 31546840 PMC6770683

[fsn33335-bib-0055] Sudhakaran, M. , & Doseff, A. I. (2020). The targeted impact of flavones on obesity‐induced inflammation and the potential synergistic role in cancer and the gut microbiota. Molecules, 25(11), 2477. 10.3390/molecules25112477 32471061 PMC7321129

[fsn33335-bib-0056] Taherkhani, S. , Suzuki, K. , & Ruhee, R. T. (2021). A brief overview of oxidative stress in adipose tissue with a therapeutic approach to taking antioxidant supplements. Antioxidants, 10(4), 594. 10.3390/antiox10040594 33924341 PMC8069597

[fsn33335-bib-0057] Tao, J. , Zhao, Y. Q. , Chi, C. F. , & Wang, B. (2018). Bioactive peptides from cartilage protein hydrolysate of spotless smoothhound and their antioxidant activity in vitro. Marine Drugs, 16(4), 100. 10.3390/md16040100 29565311 PMC5923387

[fsn33335-bib-0058] The Institute for Health Metrics and Evaluation (IHME) . (2020). The deaths that caused by high body‐mass index among all risk factors, both sexes, all ages in global . 2019.2020.10.15.

[fsn33335-bib-0059] Tiwari, P. , & Mishra, K. P. (2017). Role of flavonoids in DNA damage and carcinogenesis prevention. Journal of Carcinogenesis & Mutagenesis, 8(4), 297. 10.4172/2157-2518.1000297

[fsn33335-bib-0060] Valavanidis, A. , Vlachogianni, T. , & Fiotakis, C. (2009). 8‐Hydroxy‐2′‐deoxyguanosine (8‐OHdG): A critical biomarker of oxidative stress and carcinogenesis. Journal of Environmental Science and Health, Part C, 27(2), 120–139. 10.1080/10590500902885684 19412858

[fsn33335-bib-0061] Wang, P. , Luo, Q. , Yang, W. , Ahammed, G. J. , Ding, S. , Chen, X. , Wang, J. , Xia, X. , & Shi, K. (2021). A novel role of pipecolic acid biosynthetic pathway in drought tolerance through the antioxidant system in tomato. Antioxidants, 10(12), 1923. 10.3390/antiox10121923 34943026 PMC8750784

[fsn33335-bib-0062] Wang, W. Y. , Zhao, Y. Q. , Zhao, G. X. , Chi, C. F. , & Wang, B. (2020). Antioxidant peptides from collagen hydrolysate of redlip croaker (*Pseudosciaena polyactis*) scales: Preparation, characterization, and cytoprotective effects on H_2_O_2_‐damaged HepG2 cells. Marine Drugs, 18(3), 156. 10.3390/md18030156 32168851 PMC7142964

[fsn33335-bib-0063] Wang, Y. , Jia, J. , Ren, X. , Li, B. , & Zhang, Q. (2018). Extraction, preliminary characterization and in vitro antioxidant activity of polysaccharides from *Oudemansiella radicata* mushroom. International Journal of Biological Macromolecules, 120, 1760–1769. 10.1016/j.ijbiomac.2018.09.209 30287363

[fsn33335-bib-0064] Wang, Y. , Zhang, L. , Li, G. T. , Li, Z. H. , Dong, Z. J. , Li, Y. , & Liu, J. K. (2015). Identification and cytotoxic activities of two new trichothecenes and a new cuparane‐type sesquiterpenoid from the cultures of the mushroom *Engleromyces goetzii* . Natural Products and Bioprospecting, 5(1), 47–53. 10.1007/s13659-014-0051-1 25633363 PMC4328002

[fsn33335-bib-0065] Wang, Y. , Zhang, L. , Wang, F. , Li, Z. H. , Dong, Z. J. , & Liu, J. K. (2015). New diterpenes from cultures of the fungus *Engleromyces goetzii* and their CETP inhibitory activity. Natural Products and Bioprospecting, 5(2), 69–75. 10.1007/s13659-015-0055-5 25850378 PMC4402584

[fsn33335-bib-0066] Wiseman, H. , & Halliwell, B. (1996). Damage to DNA by reactive oxygen and nitrogen species: Role in inflammatory disease and progression to cancer. Biochemical Journal, 313(Pt 1), 17–29. 10.1042/bj3130017 8546679 PMC1216878

[fsn33335-bib-0067] Xiao, W. , & Yang, X. (2022). A key issue of national health in China: Excess energy intake and oxidative inflammation. China Journal of Chinese Materia Medica, 47, 853–861. 10.19540/j.cnki.cjcmm.20210929.601 35285183

[fsn33335-bib-0068] Xie, K. , He, X. , Chen, K. , Chen, J. , Sakao, K. , & Hou, D. X. (2019). Antioxidant properties of a traditional vine tea, *Ampelopsis grossedentata* . Antioxidants, 8(8), 295. 10.3390/antiox8080295 31395833 PMC6719964

[fsn33335-bib-0069] Yadav, D. K. , Rai, R. , Kumar, N. , Singh, S. , Misra, S. , Sharma, P. , Shaw, P. , Pérez‐Sánchez, P. , Mancera, R. L. , Choi, E. H. , Kim, M. H. , & Pratap, R. (2016). New arylated benzo[h]quinolines induce anti‐cancer activity by oxidative stress‐mediated DNA damage. Scientific Reports, 6(1), 1–13. 10.1038/srep38128 27922047 PMC5138627

[fsn33335-bib-0070] Yau, M. Y. C. , Xu, L. , Huang, C. L. , & Wong, C. M. (2018). Long non‐coding RNAs in obesity‐induced cancer. Non‐Coding RNA, 4(3), 19. 10.3390/ncrna4030019 30154386 PMC6162378

[fsn33335-bib-0071] Yunnan Institute of Botany . (1975). Fungi that produce new drugs—*Engleromyces goeti* Henn study on bamboo fungus. Plant Diversity, 1(1), 50–53 (In Chinese, without DOI code).

[fsn33335-bib-0072] Zhang, H. , Liu, R. , & Lu, Q. (2020). Separation and characterization of phenolamines and flavonoids from rape bee pollen, and comparison of their antioxidant activities and protective effects against oxidative stress. Molecules, 25(6), 1264. 10.3390/molecules25061264 32168811 PMC7144025

[fsn33335-bib-0073] Zhang, Y. , Chen, G. , Ma, H. , & Guo, M. (2019). Antiproliferative and enzyme docking analysis of engleromycin from *Engleromyces goetzei* . Molecules, 24(1), 166. 10.3390/molecules24010166 30621140 PMC6337443

[fsn33335-bib-0074] Zhishen, J. , Mengcheng, T. , & Jianming, W. (1999). The determination of flavonoid contents in mulberry and their scavenging effects on superoxide radicals. Food Chemistry, 64(4), 555–559. 10.1016/S0308-8146(98)00102-2

[fsn33335-bib-0075] Zhong, Q. , Wei, B. , Wang, S. , Ke, S. , Chen, J. , Zhang, H. , & Wang, H. (2019). The antioxidant activity of polysaccharides derived from marine organisms: An overview. Marine Drugs, 17(12), 674. 10.3390/md17120674 31795427 PMC6950075

